# On the Chemical Stability of DNA-Stabilized Silver Nanoclusters

**DOI:** 10.1021/acsomega.4c08322

**Published:** 2024-11-12

**Authors:** Giacomo Romolini, Cecilia Cerretani, Christian Brinch Mollerup, Tom Vosch

**Affiliations:** † Department of Chemistry, University of Copenhagen, Universitetsparken 5, DK-2100 Copenhagen, Denmark; ‡ Department of Forensic Medicine, University of Copenhagen, Frederik V’s Vej 11, DK-2100 Copenhagen, Denmark

## Abstract

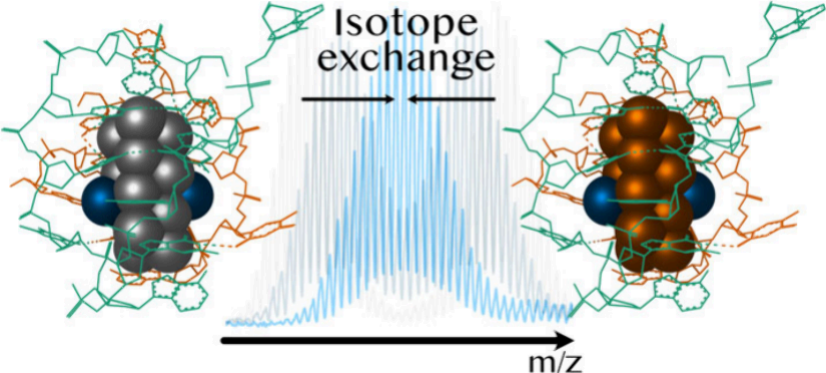

DNA-stabilized silver nanoclusters (DNA-AgNCs) are atomically precise emitters, whose chemical stability is commonly defined by their stable optical response over time. Here, two isotopically pure versions (^107^Ag and ^109^Ag) of DNA_2_-[Ag_16_Cl_2_]^8+^ were mixed, and we demonstrate for the first time for DNA-AgNCs that silver atoms are continuously exchanged between individual clusters. This atom exchange was monitored by time-resolved mass spectrometry. Depending on the temperature, the exchange happens on a time scale of minutes to hours, while DNA_2_-[Ag_16_Cl_2_]^8+^ retains its ensemble spectroscopic features. Based on the time constants and the available crystal structure, we hypothesized the following exchange mechanism: formation of transient dimers followed by atom exchange between the two entities. A further and slower exchange of the swapped Ag atoms within the rest of the clusters occurs at a much slower time scale. Our findings shed new light on the meaning of chemical stability for this class of fluorophores and show that these systems can be structurally dynamic at the molecular level, while maintaining stable ensemble spectroscopic properties. Our results also explain why DNA-AgNCs are good sensors, as the dynamic nature of DNA-AgNCs allows for competing interactions, altering their optical response.

## Introduction

DNA-stabilized silver nanoclusters (DNA-AgNCs) were first described 20 years ago by Petty et al.^1^ These emissive compounds show intriguing DNA sequence-dependent optical properties. So far, most efforts have been directed toward unravelling the role the DNA strands play in stabilizing atomically precise clusters and determining their complex photophysics. While bare clusters have been synthesized and studied in vacuum^2^ and in frozen gas matrices,^3,4^ they require ligands or scaffolds in solution^5^ and solid state^6^ to prevent aggregation and growth. Several studies have shown that DNA-AgNCs can be very stable for months and even years, when dissolved in 10 mM ammonium acetate and stored at 4 °C.^7,8^ The chemical stability of these systems is usually inferred by measuring absorption and emission spectra over time, and if no changes are observed, the DNA-AgNCs are deemed stable.

**Figure 1:**
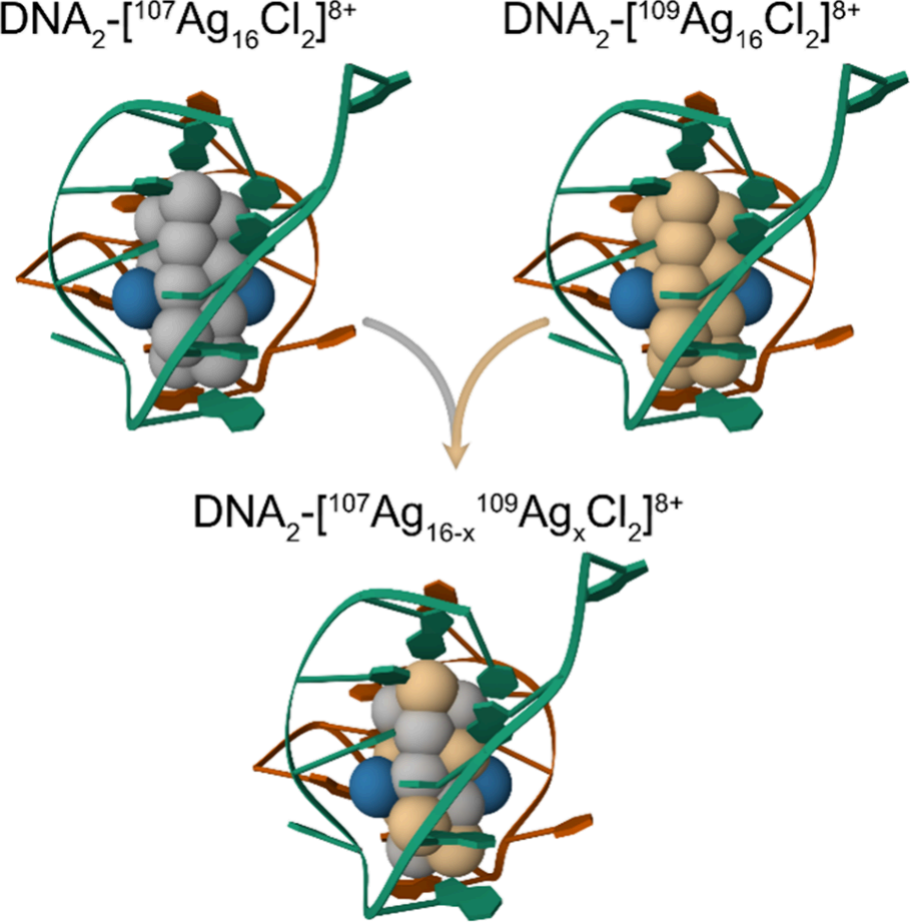
Schematic representation of the isotopic exchange after mixing isotopically pure DNA_2_-[^107^Ag_16_Cl_2_]^8+^ and DNA_2_-[^109^Ag_16_Cl_2_]^8+^. The structure was previously determined from single crystal X-ray diffraction of DNA_2_-[Ag_16_Cl_2_]^8+^ with natural Ag isotope abundancies (PDB accession code 6JR4).^9^

An example of a stable DNA-AgNC is the well-characterized NIR-emitting DNA_2_-[Ag_16_Cl_2_]^8+^. This nanocluster is the result of a reduction-induced self-assembly process, and consists of two 5′-CACCTAGCGA-3′ strands that encapsulate a 16-silver-atom rod-like cluster (see Figure 1 or PDB-ID 6JR4),^9^ which is further stabilized by two chloride ligands.^10^ The DNA provides shielding from the solvent, with only the region around the two chlorides exposed to it.^10^ Gonzàlez-Rosell et al. demonstrated that these chloride ions can be exchanged with bromide anions when an excess of NaBr is added.^10^ Moreover, the addition of bare DNA strands to DNA_2_-[Ag_16_Cl_2_]^8+^ was recently proven to lead to dramatic destabilization and destruction of the compound.^11^ This indicates that there is a dynamic competition between the added free DNA and the AgNC-stabilizing strands to bind the silver atoms and cations of the AgNC. Interestingly, addition and prebinding of a sufficient amount of silver cations to the free DNA strands preserved the chemical stability of DNA_2_-[Ag_16_Cl_2_]^8+^.

Remarkable results by Chakraborty et al.^12,13^ and Neumaier et al.^14^ showed that atomically precise AgNCs, stabilized by multiple small organic ligands could rapidly exchange atoms with each other. Chakraborty et al. demonstrated this by using equal amounts of isotopically pure AgNCs and following their exchange by mass spectrometry.^12^ Inspired by the question of what chemical stability means in the case of DNA-AgNCs, we prepared two isotopically pure versions of DNA_2_-[Ag_16_Cl_2_]^8+^, mixed them together, and monitored the exchange by mass spectrometry. Our results show that individual clusters can dynamically exchange silver atoms with each other on a time scale of minutes to hours, while maintaining their ensemble optical features.

A deeper understanding of the stabilization of the AgNCs by DNA, competition with other DNA strands and the dynamic exchange of atoms between them might enable new potential features like self-healing^15^ and regeneration.^16^ It should also allow to steer and predict the conversion of one AgNC into another with the addition of the correct templating DNA sequence.^17^ Additionally, it poses interesting questions about the meaning of “stability” for this class of materials, especially concerning the equilibria necessary to maintain the chemical stability and the desired optical response when exposed to more complex (biological) environments.

## Results and Discussion

### Spectroscopic Properties of DNA-^107^AgNC and DNA-^109^AgNC

Isotopically pure silver nitrate was synthesized from isotopically pure silver by reaction with HNO_3_. This allowed preparation of two isotopically pure starting compounds, DNA-^107^AgNC and DNA-^109^AgNC, following a procedure described previously (see the SI for details of the synthesis and HPLC purification).

Absorption and emission spectra, as well as fluorescence decay times, were measured at three different temperatures: 10, 25, and 40 °C. As shown in Table S1, fluorescence decay times, absorption and emission maxima are in line with the values reported previously for DNA_2_-[Ag_16_Cl_2_]^8+^ made with natural isotope abundances of silver (further referred to as DNA-^nat^AgNC).^7^ Moreover, we monitored the spectroscopic properties of the isotopically pure clusters, the mixture of DNA-^107^AgNC with DNA-^109^AgNC, and DNA-^nat^AgNC over time and at various temperatures. No spectroscopic changes were observed (Table S2). Additionally, for the mixture, a reversible drop in absorbance and emission intensity was found when cycling the temperature between 10 and 40 °C (Table S3). This demonstrates that the isotopically pure versions, their 1:1 mixture, and the natural DNA_2_-[Ag_16_Cl_2_]^8+^, give similar and, more importantly, stable optical responses over time at constant temperature.

### Isotope Exchange Experiment

**Figure 2:**
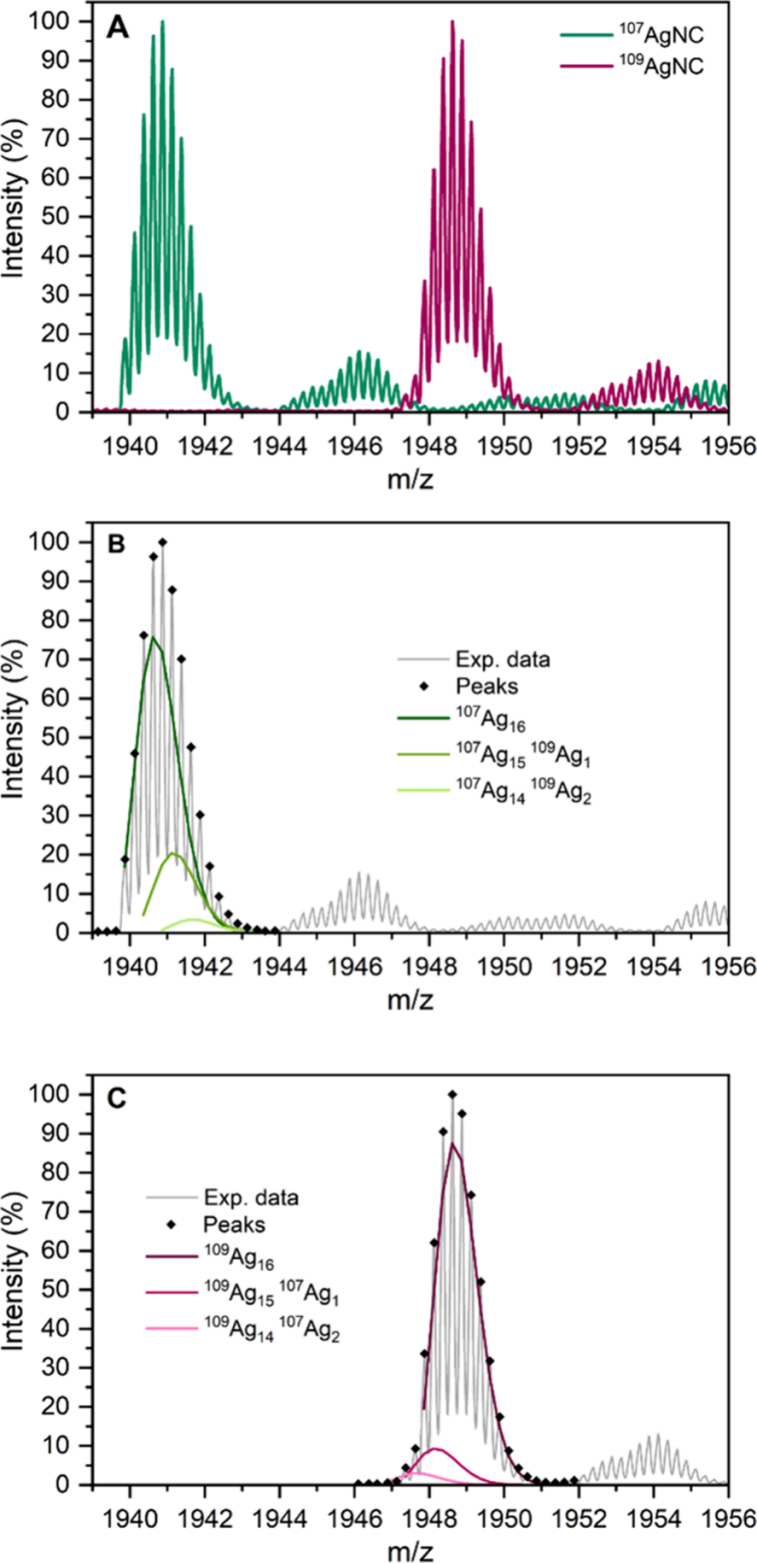
Mass spectra of DNA-^107^AgNC and DNA-^109^AgNC. (A) Overlay of the *z* = 4^–^ peaks related to DNA-^107^AgNC (green) and DNA-^109^AgNC (mauve), and deconvoluted peaks of (B) ^107^AgNC and (C) ^109^AgNC. In B, the contribution of ^107^Ag_16_, ^107^Ag_15_^109^Ag_1_, and ^107^Ag_14_^109^Ag_2_ are 75.70%, 20.43%, and 3.42%, respectively. In C, the contribution of ^109^Ag_16_, ^109^Ag_15_^107^Ag_1_, and ^109^Ag_14_^107^Ag_2_ are 87.54%, 9.31%, and 3.02%, respectively. Details on the deconvolution of the peak of the ^107^AgNC adducts can be found in Figure S10.

First, the mass spectra of DNA-^107^AgNC and DNA-^109^AgNC were measured separately (details on mass spectrometry measurements are reported in the SI). Figure 2A shows the overlay of the *z* = 4^–^ peaks related to DNA-^107^AgNC (green) and DNA-^109^AgNC (mauve). These peaks were deconvoluted to estimate the purity of the DNA-^107^AgNC and DNA-^109^AgNC solutions. Besides the main contribution coming from DNA_2_-[^107^Ag_16_Cl_2_]^8+^ or DNA_2_-[^109^Ag_16_Cl_2_]^8+^, a few percent of AgNC with one and two Ag atoms of the other isotope were found. This allowed us to calculate a purity of 97.8% and 98.8% for DNA-^107^AgNC and DNA-^109^AgNC, respectively, which are in line with the 99% isotopic purity guaranteed by the manufacturer. Furthermore, it is worth noticing that between *m*/*z* 1944 and 1948, as well as *m*/*z* 1952 and 1956 (for DNA_2_-[^107^Ag_16_Cl_2_]^8+^ and DNA_2_-[^109^Ag_16_Cl_2_]^8+^, respectively) there are minor peaks related to adducts where one or two protons were replaced by another cation like Na^+^, NH_4_^+^, or Mg^2+^ (see Figure S10). Of these three, NH_4_^+^ and Na^+^ seem to be the most plausible.

**Figure 3:**
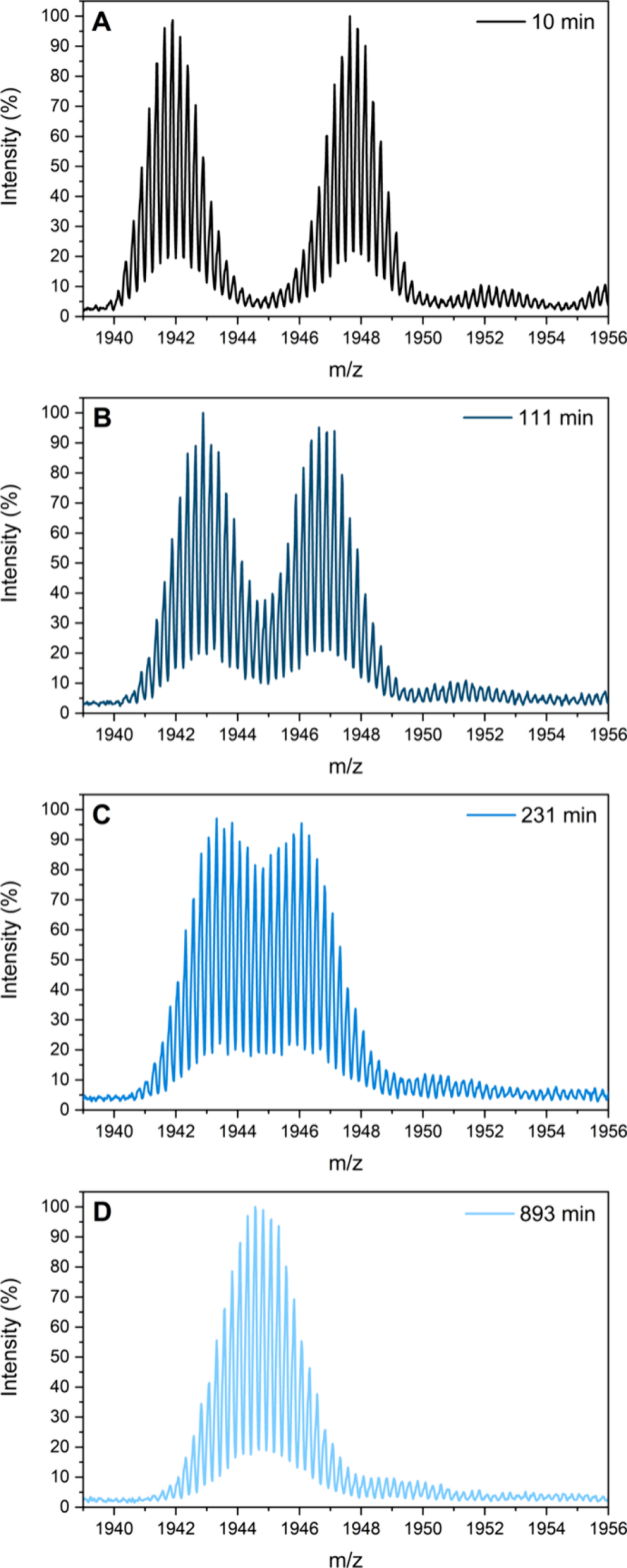
Mass spectra of the 1:1 mixture of DNA-^107^AgNC and DNA-^109^AgNC measured at 10 °C after (A) 10 min, (B) 111 min, (C) 231 min, and (D) 893 min after mixing.

Once the mass spectra of DNA-^107^AgNC and DNA-^109^AgNC were measured, equal concentrations of both were mixed together, and time-resolved mass spectrometry experiments were performed at 10, 25, and 40 °C. Mass spectra were recorded every minute for the first 10 min after mixing, and were then, depending on the temperature, acquired at different time intervals until no further evolution of the *m*/*z* peaks was observed. A few mass spectra at different time points from the 10 °C experiment can be seen in Figure 3.

At early times, two separate peaks are present (Figure 3A), which then merge together (Figure 3B, C) over time to a single peak (Figure 3D) that is approximately positioned between the original ones (see Figure 2A for comparison).

For every mass spectrum, the region related to the *z* = 4^–^ peaks was analyzed with the MSTools deconvolution software developed by Swiss Federal Institute of Technology Lausanne^18^ in order to investigate the kinetics of the isotope exchange. The mass spectra were analyzed by fitting the “envelopes” with the contribution from each of the possible DNA_2_-[^107^Ag_16–*x*_^109^Ag_*x*_Cl_2_]^8+^ (0 ≤ *x* ≤ 16) isotopologues. It is worth noting that when the isotopically pure clusters begin to exchange atoms, the ^107^AgNC adduct peak overlaps with the ^109^AgNC peak. Minor errors are thus expected when deconvoluting the ^109^AgNC peak. While this complicates the analysis of the ^109^AgNC envelope, the ^107^AgNC envelope is unaffected; hence we decided to focus only on the evolution of the ^107^AgNC envelope over time (see SI for a detailed explanation).

**Figure 4:**
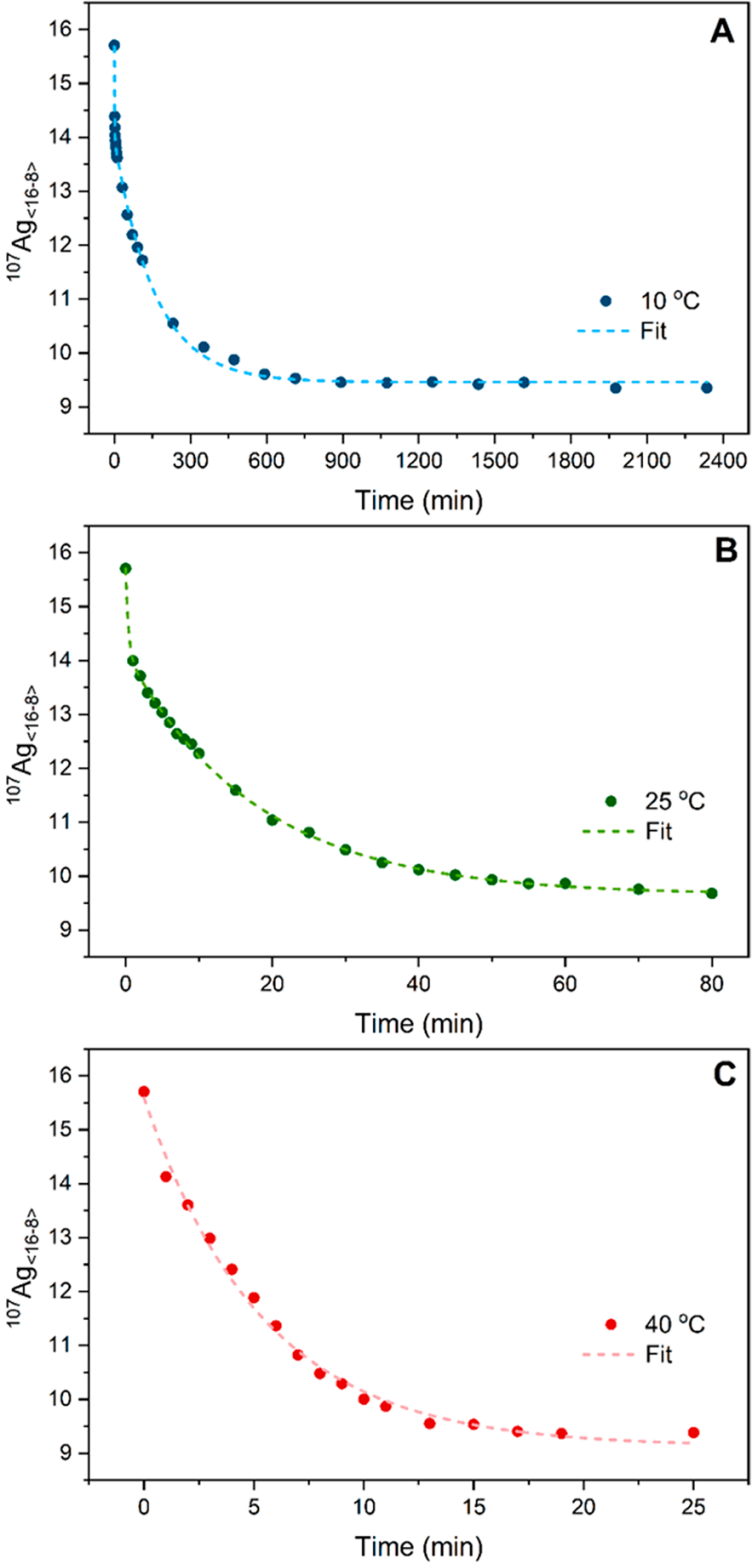
Time evolution of ^107^Ag_<16-8>_ at 10, 25, and 40 °C. The 10 and 25 °C data were fitted with biexponential decay models, while the 40 °C data was fitted with a monoexponential decay function. The point at *t* = 0 min in the three plots is taken from the data reported in Figure 2.

To better understand the exchange kinetics, we then calculated the weighted average using only the DNA_2_-[^107^Ag_16–*x*_^109^Ag_*x*_Cl_2_]^8+^ isotopologues with 0 ≤ *x* ≤ 8. (This average will be further referred to as ⟨^107^Ag_16–8_⟩). ⟨^107^Ag_16–8_⟩ equilibrates toward 9.41, with minor differences at each temperature (Figure 4). At 10 and 25 °C, the time evolution of ⟨^107^Ag_16–8_⟩ can be fitted with biexponential decay functions indicating two kinetics regimes: a very rapid exchange followed by a slower one. The 40 °C data was instead fitted with a monoexponential decay model, most likely because the very fast exchange component could not be resolved. The first time constants are comparable with the time-resolution of the measurements (0.91 and 0.37 min, for 10 and 25 °C, respectively) and might be actually smaller if a better time resolution was available. Note that the 10 °C experiment was performed twice to test the reproducibility of the isotope exchange. Figure S12 displays that the average ⟨^107^Ag_16–8_⟩ values evolve very similarly over time for both experiments, confirming the reproducibility of the exchange experiment.

**Figure 5:**
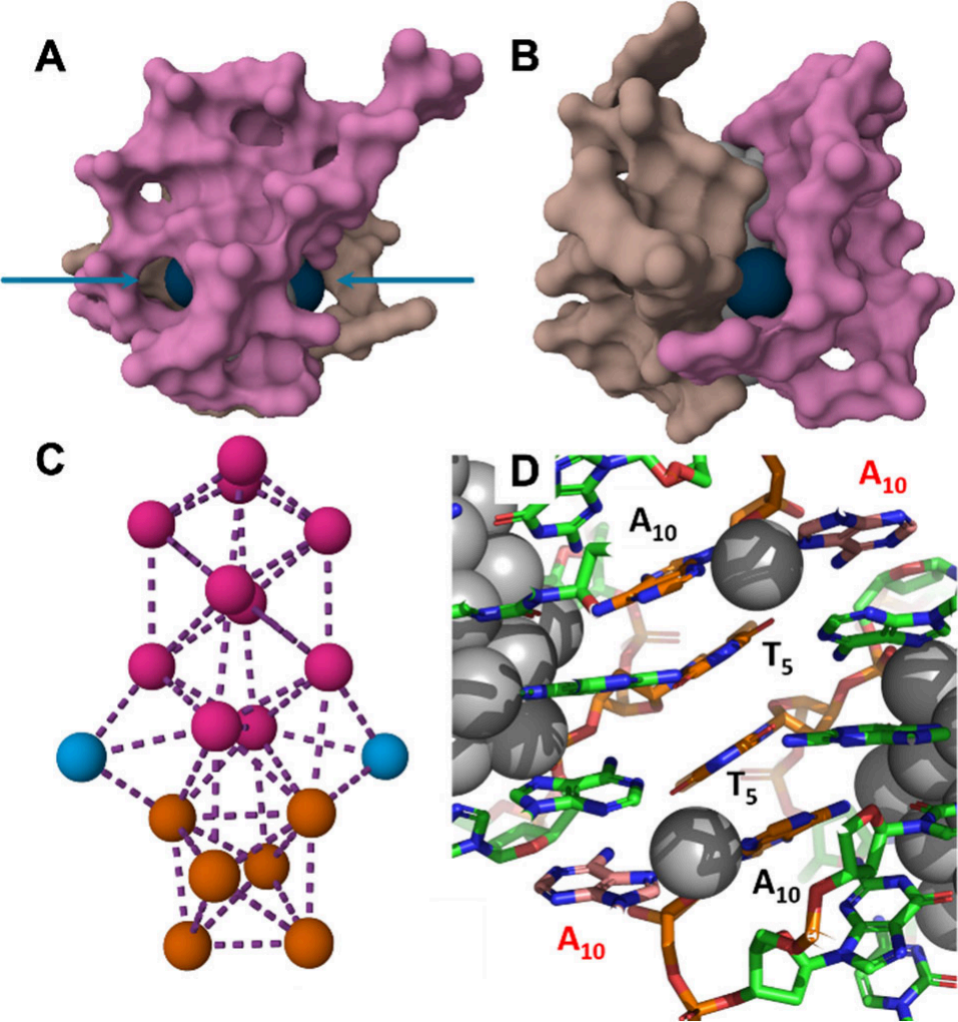
Molecular structure of DNA_2_-[Ag_16_Cl_2_]^8+^. (A and B) DNA strands are represented as surfaces highlighting the cavities around the chlorides (blue spheres). A and B are rotated 90° with respect to each other. (C) [Ag_16_Cl_2_]^8+^ part with chlorides (blue), the edge-sharing bioctahedral Ag_10_ (magenta) and the 90°-twisted Ag_6_ octahedron (orange). (D) Interactions between two DNA_2_-[Ag_16_Cl_2_]^8+^ units. The image shows the π–π stacking interactions between two thymines (T_5_) and among adenines and thymines (A_10_ and T_5_), as well as the silver-mediated adenine base pair (A_10_). Images were created based on the PDB data with accession code 6JR4.^9^

Neumaier et al. showed that the exchange of atoms between organic ligand-stabilized Ag_25_ and Au_25_ clusters can be mediated through the formation of dimers. The authors confirmed this by the appearance of an *m*/*z* peak with the mass given by the sum of both clusters and twice the charge. While no such DNA_2_-[^109^Ag_16_Cl_2_]^8+^-DNA_2_-[^107^Ag_16_Cl_2_]^8+^ dimer was observed in our mass spectrometry data, it could still be a plausible mechanism to describe the exchange of atoms between the two DNA_2_-[Ag_16_Cl_2_]^8+^ isotopologues. X-ray diffraction of single DNA-^nat^AgNC crystals^9,19−21^ shows that there is a wealth of interactions between neighboring DNA-^nat^AgNC units (See Figure 5), e.g., silver-mediated base pairs,^22^ π–π stacking and hydrogen bonds, which could also play a role in stabilizing transient dimers in solution. We postulate that, while the interactions between the dimer facilitate exchange, we do not think that a single merged entity consisting of a DNA_4_-[Ag_32_Cl_4_]^16+^ cluster is formed.

Previously, for metal clusters stabilized by small organic ligands, fast exchange rates were ascribed to the exchange of surface atoms, while slower rates were attributed to the diffusion of exchanged atoms within the cluster core.^12^ Such conceptual interpretation is not applicable in our case because DNA_2_-[Ag_16_Cl_2_]^8+^ is rod-like and all atoms are basically surface atoms bound to ligands; either to the DNA nucleobases or to the chloride anions. However, certain parts of the AgNC might still be more tightly bound to the DNA than others, explaining the different time regimes. We could ascribe the fast time constant to the exchange of silver atoms in the chloride region since this area is always exposed to the solvent environment (see Figure 5A-B). Additionally, the thymines localized close to the chlorides can interact via π–π stacking interactions and promote the formation of transient dimers. The latter were observed in the crystal structure (see Figure 5D).^9^ In solution, these interactions can be accompanied by the local unwrapping of the DNA. Another possible dimer interaction could be through silver-mediated base pairs. The crystal structure shows indeed the presence of A_10_-Ag^+^-A_10_ interactions between DNA-AgNCs of different asymmetric units (see Figure 5D).^9^ DNA-AgNCs with an additional silver cation are clearly present in solution since there is a small peak (≈10% of the base peak, Figure S11) in the mass spectrum of DNA-^nat^AgNC related to DNA_2_-[Ag_17_Cl_2_]^9+^,^10,23^ with the additional Ag^+^ most likely bound to the A_10_ nucleobase.^9^ The slower exchange regime (with time constants of 158.42 min, 17.89 and 5.41 min for 10, 25, and 40 °C, respectively) can instead be attributed to the diffusion of swapped silver atoms into the edge-sharing bioctahedral Ag_10_ part, which seems better shielded from the environment (magenta atoms in Figure 5C). It is also worth specifying that the slower exchange times could provide a more complex and heterogeneous picture of exchange at the molecular level.

**Figure 6:**
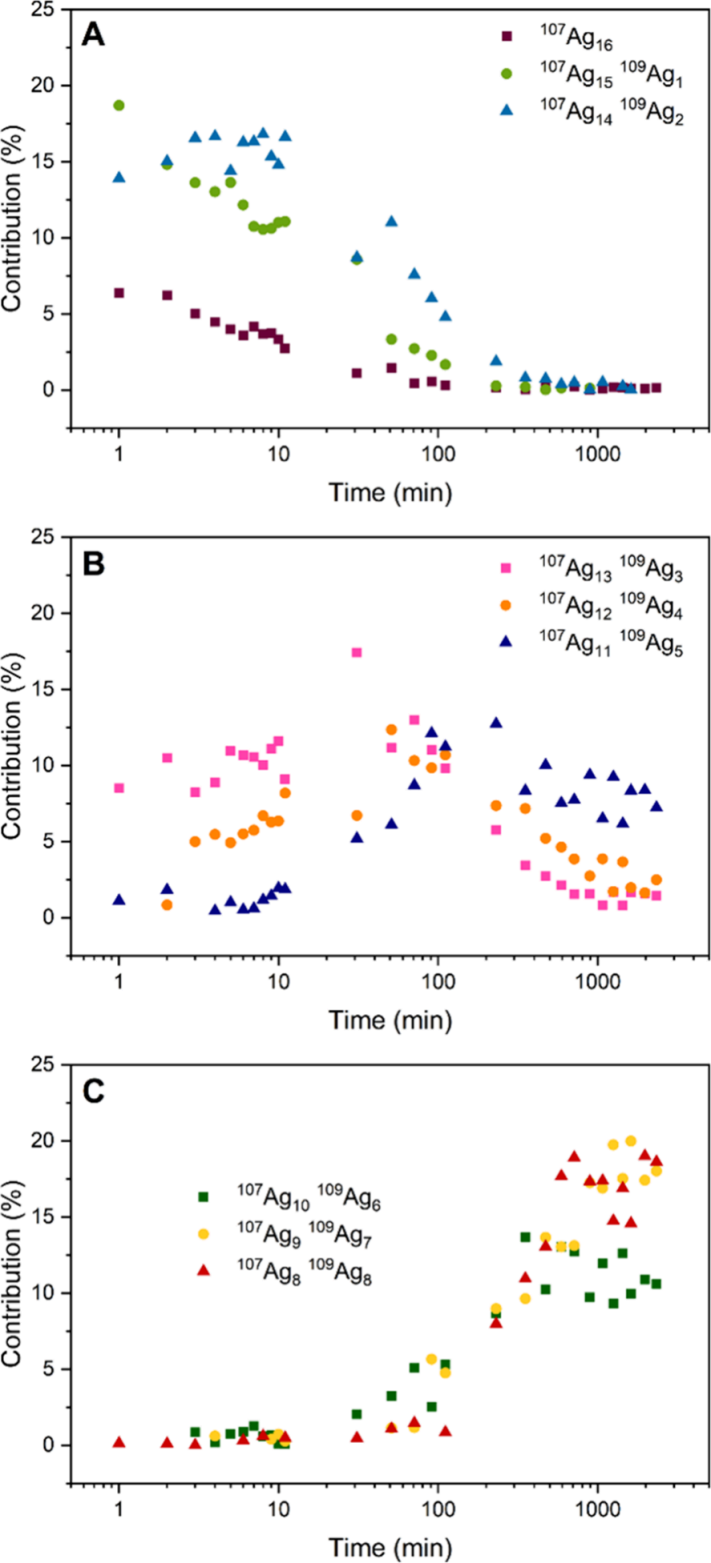
Detailed time evolution of the DNA_2_-[^107^Ag_16-x_^109^Ag_*x*_Cl_2_]^8+^ (0 ≤ *x* ≤ 8) isotopologues at 10 °C. (A) *x* = 0, 1, 2. (B) *x* = 3, 4, 5. (C) *x* = 6, 7, 8.

Figure 6 shows a more detailed view of the isotope exchange at 10 °C, with the contribution of each of the DNA_2_-[^107^Ag_16–*x*_^109^Ag_*x*_Cl_2_]^8+^ (0 ≤ *x* ≤ 8) isotopologues plotted over time (all values, including *x* > 8, can be found in Table S4). Figure 6A shows that the initial exchange of the first few atoms is very fast and only a decay in the population of the first two isotopologues, DNA_2_-[^107^Ag_16_Cl_2_]^8+^ and DNA_2_-[^107^Ag_15_^109^Ag_1_Cl_2_]^8+^ can be observed. DNA_2_-[^107^Ag_14_^109^Ag_2_Cl_2_]^8+^ follows a similar trend, albeit a small lag period is present during the first 10 min where the population is fairly constant. From the isotopologue-resolved traces, it is clear that already after 1 min the distribution is very different than that of the isotopically pure cluster (Figure 2). Figure 6B shows that the population of the next three isotopologues, DNA_2_-[^107^Ag_13_^109^Ag_3_Cl_2_]^8+^, DNA_2_-[^107^Ag_12_^109^Ag_4_Cl_2_]^8+^, and DNA_2_-[^107^Ag_11_^109^Ag_5_Cl_2_]^8+^, rises with time constants that are in the same order of magnitude to those by which the first three isotopologues disappear in Figure 6A. A maximum contribution is reached around 100 min, after which they start decaying and, as shown in Figure 6C, convert into the final three isotopologues. Figure 6C displays the very slow rise of the final three isotopologues, DNA_2_-[^107^Ag_10_^109^Ag_6_Cl_2_]^8+^, DNA_2_-[^107^Ag_9_^109^Ag_7_Cl_2_]^8+^, and DNA_2_-[^107^Ag_8_^109^Ag_8_Cl_2_]^8+^, which take over 1000 min to reach their equilibrated state.

Despite the bulky DNA scaffold and “stable” photophysical ensemble response, our experiments demonstrate that DNA_2_-[Ag_16_Cl_2_]^8+^ is structurally dynamic at the molecular level. We propose here a model that, when two DNA-AgNCs meet, a transient dimer is formed, allowing for atom exchange. Afterward, the dimer dissociates in two monomers with a different isotope distribution, while the overall [Ag_16_Cl_2_]^8+^ composition is maintained. From an ensemble point of view, entropy can be considered the driving force for the isotopic exchange.^12^ At the molecular level, we can instead hypothesize that intermolecular interactions between DNA_2_-[Ag_16_Cl_2_]^8+^ units steer the atom exchange, meaning that the atom swap will continue even when the silver isotope distribution has equilibrated. We note that our model is speculative at this point, and below we discuss some alternative exchange mechanisms that we believe are less likely. Another model for the isotope exchange could be that the DNA_2_-[Ag_16_Cl_2_]^8+^ splits in half and the resulting segments meet with their isotope counterparts to form isotopically mixed DNA_2_-[Ag_16_Cl_2_]^8+^. While it has been shown for another DNA-AgNC that it can be formed by using two halves of an 18-base DNA sequence, the presence of split clusters would normally result in diverse absorption features.^24,25^ Since the absorption and fluorescence excitation spectra do not differ, it seems unlikely that this would be the leading cause of isotope exchange. Moreover, this would lead to the presence of fully exchanged isotopologues from the beginning, yielding a different kinetic exchange profile and not the gradual exchange observed in Figure 6. Another exchange mechanism can be mediated by free silver cations in solution that can bind and exchange with the core atoms of DNA_2_-[Ag_16_Cl_2_]^8+^. While this is a very efficient way of swapping atoms, the isotopically pure samples were HPLC-purified and solvent exchanged to remove any free silver cations. The mass spectrometry data in Figure S11 shows that the presence of the compound DNA_2_-[Ag_17_Cl_2_]^9+^ (i.e., with one additional Ag^+^ compared to the main product) is around 10%, which makes us believe that the potential amount of free silver cations is most likely too low to be the dominant exchange mechanism. However, we can not fully exclude that this mechanism is relevant here.

## Conclusion

Our results provide a framework to explain how DNA-AgNCs can be considered chemically stable in solution at the ensemble level and yet can be very dynamic from a molecular point of view. We demonstrated this by following the exchange of silver isotopes between DNA-^107^AgNC and DNA-^109^AgNC using time-resolved mass spectrometry. Given the favorable intermolecular interactions observed in the crystalline state, we postulate that the isotope exchange occurs through the formation of transient dimers. The dynamic exchange of silver atoms does not affect the ensemble optical response, which remains stable and unaltered. However, as illustrated by many examples in the literature, it is now apparent that the presence of free DNA strands or other species in solution affects this equilibrium, leading to the great sensing capabilities of DNA-AgNCs. Finally, our results open up new avenues for controlled conversion from one DNA-AgNC to another and the creation of self-healing DNA-AgNCs.

## Supporting Information Available

Materials and methods section, steady-state and time-resolved photophysical data and additional ESI-MS data (PDF)
