# Supplementary material for: On the Chemical Stability of DNA-Stabilized Silver Nanoclusters
Source: ACS Omega. 2024 Nov 12;9(47):47248–53. doi: 10.1021/acsomega.4c08322 (PMC11603216; doi:10.1021/acsomega.4c08322)
Supplement: Supplementary file 1 — ao4c08322_si_001.pdf [file ao4c08322_si_001.pdf]

Supporting Information

On the chemical stability of DNA-stabilized silver nanoclusters.

Giacomo Romolini,[a] Cecilia Cerretani,[a,*] Christian Brinch Mollerup,[b] Tom Vosch[a,*]

[a] *Department of Chemistry, University of Copenhagen, Universitetsparken 5, DK-2100 Copenhagen, Denmark.*
[b] *Department of Forensic Medicine, University of Copenhagen, Frederik V's Vej 11, DK-2100 Copenhagen, Denmark.*

1. Materials

The DNA oligonucleotide with the sequence 5'-CACCTAGCGA-3′ and nuclease-free water were purchased from Integrated DNA Technologies.
Natural $AgNO_3$ (≥ 99.998%), ammonium acetate (≥ 98%) and $NaBH_4$ (≥ 99.99%) were acquired from Sigma Aldrich.
HNO_3 (> 68%) trace analysis-grade was purchased from Thermo Fisher Scientific.
50 mg of each isotope, either in the form of metal powder (^{109}Ag) or metal ingot (^{107}Ag), were bought from Neonest AB.

2. Syntheses and HPLC purification

<u>2.1 Syntheses of isotopically pure $AgNO_3$ salts</u>
50 mg of each isotope, either in the form of metal powder (^{109}Ag) or metal ingot (^{107}Ag), were reacted independently with approximately 3 mL of 68% HNO_3 while heating at 70 ˚C overnight. An excess amount of HNO_3 was used to ensure the complete dissolution of the metals. The following day, the solutions appeared colorless, indicating that the solids had fully dissolved. The solutions were then diluted twice, each time with 1 mL of Milli-Q H_2O, and left open to allow the water to slowly evaporate. The resulting saturated solutions were then transferred to a desiccator until dry white crystals formed. The crystals were then used to synthesize isotopically pure DNA-AgNCs.

<u>2.2 Syntheses of DNA_2-$[Ag_{16}Cl_2]^{8+}$ nanoclusters</u>
The syntheses of isotopically pure DNA-^{107}AgNC, DNA-^{109}AgNC and DNA-^{nat}AgNC (*i.e.*, with natural silver isotope abundances) were performed using the procedure reported by Bogh *et al.*[1] The final concentrations were as follows: 25 μM DNA, 187.5 μM $^{107}AgNO_3$, $^{109}AgNO_3$ or $^{nat}AgNO_3$, 93.75 μM $NaBH_4$, and 10 mM ammonium acetate (NH_4OAc) with pH 7. The solutions were left to react for 3 days at 4 ˚C. On the third day, the two solutions were upconcentrated with 3 kDa cut-off membrane centrifugal filters (Amicon Ultracel-3) and then purified through HPLC. Before running the purification, the HPLC column was thoroughly washed with a mixture of 60% 0.1 M HNO_3 (in Milli-Q H_2O) and 40% methanol for 1 hour to ensure no contamination from residual silver-containing products. Then, the column was washed with a mixture of 60% methanol and 40% Milli-Q H_2O to remove any remaining nitrate compounds.

<u>2.3 HPLC purification</u>
HPLC purification was performed using a preparative HPLC system from Agilent Technologies with an Agilent Technologies 1200 Series UV-Vis detector, an Agilent Technologies 1100 Series fluorescence detector, and a Kinetex C18 column (Phenomenex, 5 μm, 100 Å, 250 × 4.6 mm), equipped with a fraction collector (Agilent Technologies 1200 Series), a binary pump (Agilent

Technologies 1200 Series) and an injector module (Agilent Technologies 1260 Infinity). The mobile phase was a gradient mixture of 35 mM triethylammonium acetate (TEAA) buffer in water (A) and methanol (B). The flow rate was set to 1 mL/min.

In the first 2 min the elution gradient was kept constant at 25% B, then it was linearly increased to 41% B in the next 16 min, and finally in the 18-20 min interval, the gradient was rapidly risen to 95% B. The collection was based on the absorbance at 530 nm. Additionally, the AgNC emission band was monitored at 730 nm, exciting at 530 nm. The run was followed by 3 min of washing with 95% B to remove any remaining sample from the column. The chromatograms can be found in Figures S3 and S4.

The collected fractions were then solvent-exchanged with 10 mM NH_4OAc using 3 kDa cut-off membrane centrifugal filters (Amicon Ultracel-3).

3. Spectroscopic measurements

All spectroscopic measurements were performed for both isotopically pure DNA-^{107}AgNC and DNA-^{109}AgNC, and DNA-^{nat}AgNC. The collected spectra and decay curves are shown in Figures S5-S9, while the absorption and emission maxima together with the intensity-weighted average decay times are reported in Tables S1-S3.

3.1 Absorption measurements

Absorption spectra were measured with a Cary 300 UV-Vis spectrophotometer from Agilent Technologies using a deuterium lamp for ultraviolet radiation and a tungsten-halogen lamp for visible and near-infrared (NIR) radiation. All measurements were performed in a single-beam configuration with a “zero/baseline” correction, *i.e.*, measuring the 100%/0% transmittance with air as reference. The corresponding solvent spectra were measured separately and then subtracted from the samples’ spectra. The absorbance of the AgNC-related transition was kept below 0.1 to avoid inner filter effects during emission measurements.

All absorption spectra were measured at 10, 25 and 40 ˚C by controlling the temperature with a Cary single cell Peltier accessory.

3.2 Steady-state emission measurements

Steady-state fluorescence measurements were performed using a FluoTime300 instrument (PicoQuant). The fluorescence spectra were recorded at 10, 25 and 40 ˚C by exciting the samples with a vertically-polarized 531-nm picosecond-pulsed laser (LDH-D-TA-530B, PicoQuant). All emission spectra have been corrected for the wavelength dependency of the detector.

3.3 Time-correlated single photon counting (TCSPC) measurements

Time-resolved fluorescence measurements were carried out on a FluoTime300 instrument (PicoQuant) at the above-mentioned temperatures by exciting with a vertically-polarized 531-nm picosecond-pulsed laser (LDH-D-TA-530B, PicoQuant). The repetition rate of the laser was set to either 12.8 or 20 MHz.

Fluorescence decays were acquired at 730 nm integrating 20-30 s to reach at least 10,000 counts at the maximum. The decay curves were fitted with FluoFit v.4.6 software from PicoQuant, using a biexponential reconvolution model including the instrument response function (IRF).

3.4 Spectroscopic investigation of a 1:1 mixture of DNA-^{107}AgNC and DNA-^{109}AgNC

A 1:1 mixture of DNA-^{107}AgNC and DNA-^{109}AgNC was monitored over time by measuring absorption and emission spectra, along with fluorescence decays. All spectroscopic measurements were performed as described above, at different times: right after mixing the two isotopically pure DNA-AgNCs (5 min), then after 60, 120 and 180 min.

3.5 Temperature cycle experiment

Absorption and emission spectra together with fluorescence decays of a 1:1 mixture of DNA-^{107}AgNC and DNA-^{109}AgNC were measured at 10 ˚C, 25 ˚C, 40 ˚C and cycled back at 25 ˚C first and then at 10 ˚C. All the measurements were performed as explained above. The spectra and decay curves are shown in Figure S9, while the absorption and emission maxima and the intensity-weighted average decay times are reported in Table S3.

4. Electrospray ionization-Mass Spectrometry (ESI-MS)

4.1 Mass Spectrometry measurements

ESI-MS measurements were performed with a Xevo G2-XS QTof (Waters Corporation, Milford, MA, USA), using negative ion mode with a 2 kV capillary voltage, 30 V cone voltage and no collision energy. Spectra were collected from *m/z* 750 to 4000, with a scan time of 1 s. Source temperature was 80 ˚C with a cone gas flow of 45 L/h, the desolvation temperature and gas flow were 150 ˚C and 450 L/h, respectively. The QTOF was calibrated using ESI-L Low Tune Mix (Agilent Technologies, Santa Clara, CA, USA), which contained compounds in the mass range of *m/z* 1034 to 2834 for negative mode. All samples were injected using an Acquity I-Class Plus system (Waters) with a flow-through needle autosampler. The flow of 50 mM NH_4Oac buffer at pH 7 – MeOH (80:20) was set to 0.1 mL/min, using 3 μL injection volume. The system was operated using UNIFI v.1.9.4 (Waters), and the final spectra were generated by averaging multiple spectra surrounding the apex of the observed peak. Before injection and mixing, the samples were placed in the sample holder set to the target temperature (10 ˚C, 25 ˚C, or 40 ˚C) for 20 min and kept constant throughout all measurements.

4.2 Data analysis

For every mass spectrum, the region related to the $z=4^-$ peaks (*i.e.*, between *m/z* 1939 and 1951) was first analyzed with a self-written MatLab program to find the local maxima of each isotope envelope. An example is provided below.

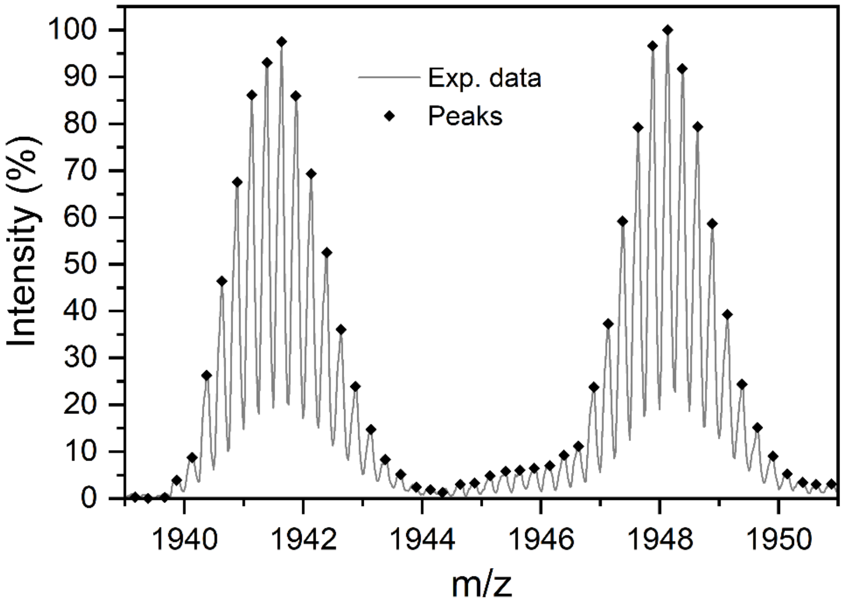

Figure S1. Example of extraction of peak maxima with the self-written MatLab script, t=1 min at 10 ˚C.

Afterwards, the peaks identified by the MatLab program were uploaded in MSTools deconvolution software developed by EPFL (https://mstools.epfl.ch/deconvolution/), and analyzed as follows.
First, the software generates the single theoretical mass spectra of $DNA_2\text{-}[^{107}Ag_{16-x}{}^{109}Ag_xCl_2]^{8+}$ ($0 \leq x \leq 16$). It then deconvolutes the experimental spectrum through a non-negative matrix factorization

algorithm. The output of the non-negative matrix factorization algorithm gives the relative contributions (weights) of the individual mass spectra of DNA_2-[$^{107}Ag_{16-x}{}^{109}Ag_xCl_2$]$^{8+}$ ($0 \leq x \leq 16$) to the experimental spectrum. The relative contributions are presented as percentages in Table S4.
Finally, to obtain the average composition of the cluster at each time, we calculated the weighted average number of ^{107}Ag atoms using the weights (%) obtained from the software output.
Table S4 clearly shows that, unlike for the $^{107}Ag_{16}$ isotopologue, the contribution of the $^{109}Ag_{16}$ isotopologue does not drop to 0 after equilibration (t=2336 min) but stabilizes at around 3.5%. This is of course not possible and is due to the following reason. In the *m/z* 1944-1948 region at t=0 min, there is a minor peak related to ^{107}AgNC adducts (see Figures 2 and S10). As the isotope exchange proceeds, this peak moves to higher *m/z* values and overlays with the ^{109}AgNC peak. This distorts the real weight of all the $^{107}Ag_x{}^{109}Ag_{16-x}$ ($0 \leq x \leq 7$) species, because the software mistakenly attributes some of ^{107}AgNC adducts as $^{107}Ag_x{}^{109}Ag_{16-x}$ ($0 \leq x \leq 7$) species. Hence, there are minor errors in the reported contributions, both in the manuscript and Table S4-S5, but we estimate them to be 10% maximum. This is the reason why, when calculating the weighted average, we used only the $^{107}Ag_x{}^{109}Ag_{16-x}$ ($8 \leq x \leq 16$) isotopologues.
It is important to mention that in our analysis we opted for not including contributions of the ^{107}AgNC adducts. This would have increased the complexity of the analysis, resulting in too many degrees of freedom and a non-meaningful outcome.

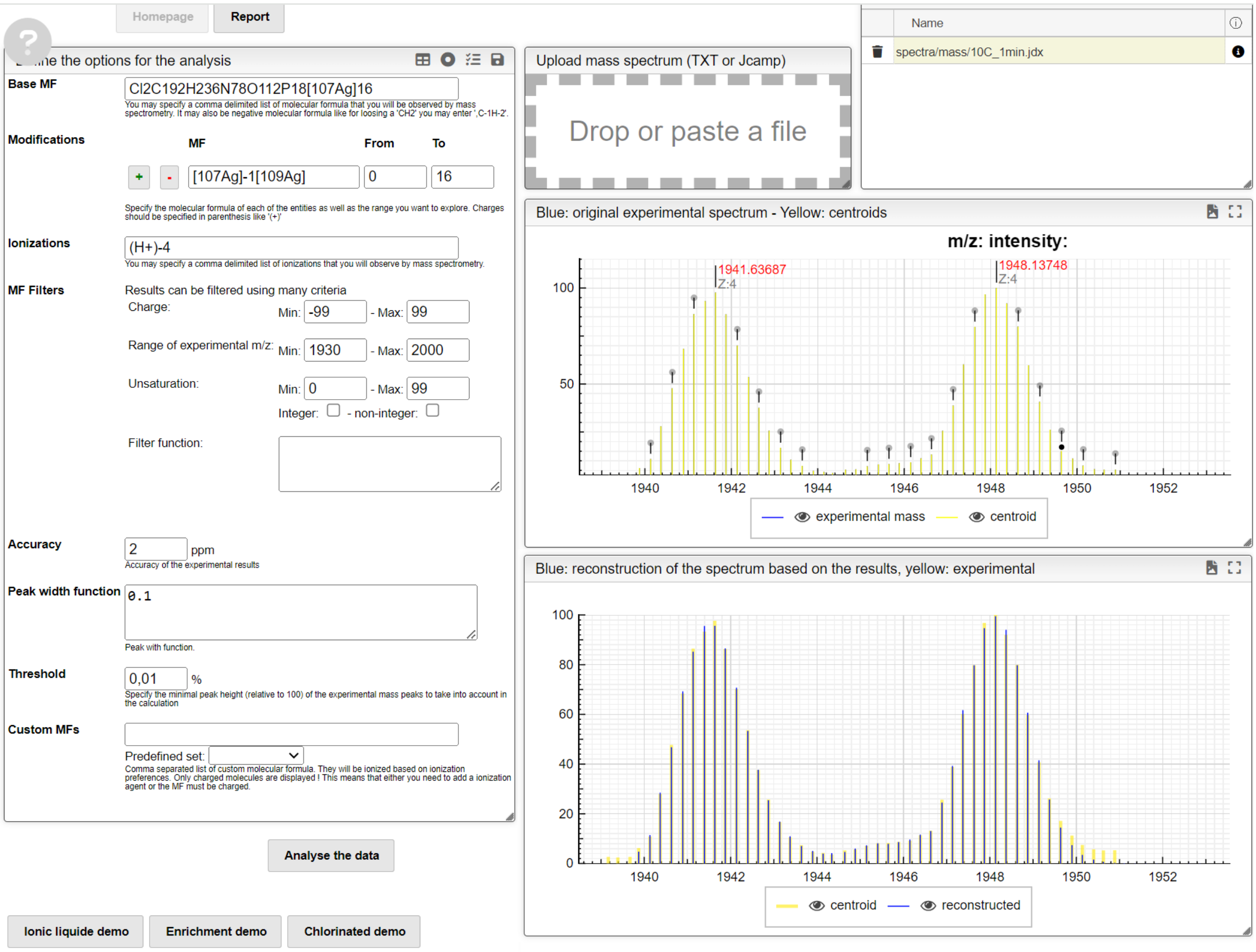

Figure S2. Example of deconvolution analysis by MSTools deconvolution software (https://mstools.epfl.ch/deconvolution/).

5. Tables and Graphs

	Temperature (˚C)	λ_{abs} (nm)	λ_{em} (nm)	<τ> (ns)
**DNA-^{nat}AgNC**	10	525	735	3.60
	25	525	735	3.24
	40	525	735	2.81
**DNA-^{107}AgNC**	10	525	729	3.53
	25	525	729	3.13
	40	526	739	2.70
**DNA-^{109}AgNC**	10	525	733	3.54
	25	525	734	3.13
	40	526	734	2.71

Table S1. Spectroscopic properties of the natural DNA_2-$[Ag_{16}Cl_2]^{8+}$ and the two isotopically pure versions at different temperatures. The emission spectra were recorded exciting at 531 nm. The fluorescence decays were measured at 730 nm, exciting at 531 nm. See Figures S5, S6 and S7 for data.

	Time (min)	λ_{abs} (nm)	λ_{em} (nm)	<τ> (ns)
**1:1 mixture (25 °C)**	5	525	735	3.13
	60	525	733	3.15
	120	525	733	3.15
	180	525	733	3.14

Table S2. Spectroscopic properties of the 1:1 mixture of DNA-^{107}AgNC and DNA-^{109}AgNC at 25 ˚C. The emission spectra were recorded exciting at 531 nm. The fluorescence decays were measured at 730 nm, exciting at 531 nm. See Figure S8 for data.

	Temperature (˚C)	λ_{abs} (nm)	λ_{em} (nm)	<τ> (ns)
**1:1 mixture**	10	526	734	3.52
	25	526	733	3.14
	40	526	734	2.68
	25 (II)	527	733	3.14
	10 (II)	525	733	3.54

Table S3. Spectroscopic properties of the 1:1 mixture of DNA-^{107}AgNC and DNA-^{109}AgNC at different temperatures. The emission spectra were recorded exciting at 531 nm. The fluorescence decays were measured at 730 nm, exciting at 531 nm. See Figure S9 for data.

Time (min)	$^{107}Ag_{16}$ $^{109}Ag_0$ (%)	$^{107}Ag_{15}$ $^{109}Ag_1$ (%)	$^{107}Ag_{14}$ $^{109}Ag_2$ (%)	$^{107}Ag_{13}$ $^{109}Ag_3$ (%)	$^{107}Ag_{12}$ $^{109}Ag_4$ (%)	$^{107}Ag_{11}$ $^{109}Ag_5$ (%)	$^{107}Ag_{10}$ $^{109}Ag_6$ (%)	$^{107}Ag_9$ $^{109}Ag_7$ (%)	$^{107}Ag_8$ $^{109}Ag_8$ (%)
**1**	6.37	18.70	13.91	8.50	0	1.11	0	0	0.13
**2**	6.21	14.82	15.02	10.50	0.83	1.82	0	0	0.11
**3**	5.01	13.62	16.57	8.24	4.99	0	0.86	0	0.01
**4**	4.48	13.03	16.66	8.89	5.47	0.44	0.20	0.61	0

**5**	3.99	13.63	14.40	10.97	4.93	1.00	0.76	0	0
**6**	3.58	12.16	16.28	10.68	5.51	0.52	0.90	0	0.33
**7**	4.17	10.75	16.32	10.56	5.75	0.60	1.27	0	0
**8**	3.69	10.55	16.81	10.03	6.70	1.16	0.58	0	0.62
**9**	3.74	10.62	15.35	11.11	6.28	1.44	0.68	0.40	0
**10**	3.34	11.02	14.80	11.59	6.35	1.94	0.11	0.73	0
**11**	2.73	11.07	16.63	9.10	8.19	1.87	0.08	0.27	0.50
**31**	1.11	8.58	8.71	17.42	6.72	5.20	2.05	0	0.47
**51**	1.47	3.33	11.01	11.15	12.36	6.11	3.23	1.14	1.09
**71**	0.43	2.72	7.57	12.99	10.32	8.70	5.08	1.17	1.47
**91**	0.55	2.28	6.03	11.03	9.84	12.12	2.55	5.66	0
**111**	0.31	1.67	4.79	9.82	10.70	11.23	5.32	4.76	0.86
**231**	0.16	0.27	1.87	5.77	7.37	12.73	8.68	8.98	7.98
**352**	0.04	0.20	0.80	3.43	7.17	8.35	13.67	9.64	10.96
**472**	0.20	0.02	0.73	2.72	5.21	10.02	10.24	13.66	13.06
**592**	0	0.13	0.38	2.14	4.64	7.54	13.06	13.05	17.69
**713**	0.21	0	0.49	1.55	3.86	7.74	12.72	13.12	18.91
**893**	0.03	0.12	0.01	1.56	2.74	9.38	9.72	17.25	17.34
**1074**	0.11	0	0.51	0.82	3.87	6.51	11.96	16.9	17.39
**1254**	0.19	0	0	1.69	1.72	9.25	9.32	19.75	14.76
**1435**	0.16	0	0.25	0.8	3.66	6.18	12.63	17.53	16.9
**1615**	0.12	0	0.03	1.67	1.97	8.35	9.96	19.99	14.58
**1976**	0.1	0	0	1.57	1.63	8.38	10.91	17.42	19.01
**2336**	0.13	0	0	1.44	2.49	7.24	10.63	18.03	18.63

**Time (min)**	**$^{107}Ag_7$ $^{109}Ag_9$ (%)**	**$^{107}Ag_6$ $^{109}Ag_{10}$ (%)**	**$^{107}Ag_5$ $^{109}Ag_{11}$ (%)**	**$^{107}Ag_4$ $^{109}Ag_{12}$ (%)**	**$^{107}Ag_3$ $^{109}Ag_{13}$ (%)**	**$^{107}Ag_2$ $^{109}Ag_{14}$ (%)**	**$^{107}Ag_1$ $^{109}Ag_{15}$ (%)**	**$^{107}Ag_0$ $^{109}Ag_{16}$ (%)**
**1**	1.13	1.00	0.42	1.94	2.69	17.08	16.96	10.05
**2**	1.13	0.31	1.72	1.86	6.47	16.71	15.12	7.37
**3**	0	2.42	0	4.01	6.33	18.51	12.41	7.02
**4**	0.61	0.77	2.26	2.13	10.17	15.06	13.41	5.82
**5**	0.82	0.74	2.27	2.34	10.68	15.36	12.32	5.79
**6**	0.31	1.43	1.19	4.74	9.23	16.45	11.81	4.87
**7**	0.73	1.08	2.11	4.68	9.89	15.17	12.04	4.87
**8**	0.07	2.07	0.77	6.09	7.68	20.13	7.07	5.99
**9**	0.56	1.43	1.72	4.97	11.66	14.23	11.51	4.30
**10**	0.24	1.88	0.93	6.97	9.04	17.05	9.04	4.96
**11**	0	1.67	1.42	6.49	10.60	15.66	9.36	4.38
**31**	1.44	0.02	6.37	8.00	12.61	13.33	5.43	2.54
**51**	1.10	2.81	6.00	10.92	12.82	9.56	3.82	2.09
**71**	1.16	5.71	6.02	15.15	7.26	11.9	0	2.34
**91**	3.12	6.18	7.78	13.85	6.74	9.77	0	2.49
**111**	5.05	4.30	12.41	9.87	9.98	6.54	0	2.40

**231**	6.20	12.05	7.73	10.80	3.49	3.72	0	2.20
**352**	12.41	9.42	10.38	5.09	4.33	1.57	0	2.56
**472**	13.34	9.87	9.79	2.84	4.71	0.35	0	3.24
**592**	12.43	9.62	11.15	0.03	5.04	0.01	0	3.08
**713**	9.24	17.3	3.87	4.98	1.62	0.89	0	3.49
**893**	13.41	14.72	3.47	4.83	1.37	0.80	0	3.24
**1074**	16.00	10.48	7.08	2.07	2.37	0.27	0	3.65
**1254**	19.75	7.65	8.9	0	3.7	0	0	3.31
**1435**	16.81	11	6.09	2.03	2.37	0.29	0	3.34
**1615**	19.67	8.2	7.19	2.59	1.48	0.6	0	3.59
**1976**	15.57	11.69	6.36	1.44	2.61	0	0	3.31
**2336**	16.13	11.37	6.27	1.69	2.41	0.23	0	3.31

Table S4. First mass spectrometry measurements performed at 10 ˚C. First part: evolution of the DNA_2-[$^{107}Ag_{16-x}{}^{109}Ag_xCl_2]^{8+}$ ($0 \leq x \leq 8$) isotopologues over time. Second part: evolution of the DNA_2-[$^{107}Ag_{16-x}{}^{109}Ag_xCl_2]^{8+}$ ($9 \leq x \leq 16$) isotopologues over time.

Time (min)	$^{107}Ag_{16}$ $^{109}Ag_0$ (%)	$^{107}Ag_{15}$ $^{109}Ag_1$ (%)	$^{107}Ag_{14}$ $^{109}Ag_2$ (%)	$^{107}Ag_{13}$ $^{109}Ag_3$ (%)	$^{107}Ag_{12}$ $^{109}Ag_4$ (%)	$^{107}Ag_{11}$ $^{109}Ag_5$ (%)	$^{107}Ag_{10}$ $^{109}Ag_6$ (%)	$^{107}Ag_9$ $^{109}Ag_7$ (%)	$^{107}Ag_8$ $^{109}Ag_8$ (%)
**1**	16.18	21.65	15.96	3.11	1.94	0	0	0.34	0.31
**2**	11.37	18.87	14.7	7.15	0.57	1.27	0	0	0.53
**3**	8.31	15.75	15.38	7.94	2.37	0.55	0.46	0	0.39
**4**	7.08	14.61	15.79	7.79	2.75	1.48	0	0	0.75
**5**	5.3	12.16	15.7	8.59	3.58	1.57	0	0.35	0.21
**6**	4.96	11.82	15.31	8.9	4.23	1.28	0	0.7	0
**7**	5.33	11.54	15.75	8.99	4.25	1.52	0	0.6	0
**8**	4.45	11.13	14.99	9.76	4.6	1.27	0.63	0	0.59
**9**	4.11	10.95	14.02	10.9	4.6	1.42	0.81	0	0.39
**10**	4.08	11.22	14.81	10.55	4.35	2.14	0	0.51	0.19
**15**	3.81	9.57	15.34	10.76	6.08	1.67	1.03	0	0.55
**20**	2.99	7.86	14.51	11	6.62	3.31	0.73	0.09	0.86
**25**	2.38	6.59	12.76	11.12	8.96	2.42	1.49	0.49	0.56
**30**	2	4.7	12.34	10.47	9.57	3.18	2.33	0	1.04
**35**	1.57	4.37	10.67	10.3	10.19	4.46	1.93	0.85	0.61
**40**	1.39	3.37	9.29	9.97	11.01	4.37	3.22	0.72	0.25
**45**	1.33	2.5	8.41	9.95	10.08	5.88	2.97	1.07	0.84
**50**	1.07	2.09	7.7	9.76	9.1	7.72	2.34	1.68	0.62
**55**	1.02	1.68	6.85	8.96	9.88	7.49	2.98	2.12	0
**60**	0.92	1.57	6.24	8.64	10.23	7.11	3.49	2.25	0.04
**70**	0.91	1.15	5.74	8.05	10.14	7.84	3.87	2.59	0
**263**	0.72	0	2.5	5.34	7.37	10.1	8.67	7.76	4.55
**443**	0	0	0.76	2.52	4.38	7.7	9.62	9.82	8.98
**624**	0	0	0.5	1.6	3.41	6.44	9.34	11.85	12.27

**804**	0	0	0.32	1.21	2.48	6.21	8.94	12.81	13.96
**1367**	0	0	0.2	1.05	2.55	6.14	10.66	14.5	18.55
**2400**	0	0	0.17	1.34	2.46	6.69	10.7	17.06	16.2

**Time (min)**	^{107}**Ag$_7$** ^{109}**Ag$_9$** **(%)**	^{107}**Ag$_6$** ^{109}**Ag$_{10}$** **(%)**	^{107}**Ag$_5$** ^{109}**Ag$_{11}$** **(%)**	^{107}**Ag$_4$** ^{109}**Ag$_{12}$** **(%)**	^{107}**Ag$_3$** ^{109}**Ag$_{13}$** **(%)**	^{107}**Ag$_2$** ^{109}**Ag$_{14}$** **(%)**	^{107}**Ag$_1$** ^{109}**Ag$_{15}$** **(%)**	^{107}**Ag$_0$** ^{109}**Ag$_{16}$** **(%)**
**1**	1.4	1.69	2.03	5.81	5.55	14.6	4.67	4.74
**2**	1.05	1.52	2.14	4.79	8.05	13.79	8.85	5.34
**3**	0.9	1.68	0.55	5.39	8.13	14.73	10.84	5.68
**4**	0.36	1.91	1.26	6.02	7.79	16.52	9.91	5.98
**5**	0.72	1.38	1.58	5.55	8.81	16.36	12.16	5.97
**6**	0.78	1.27	1.75	5.84	8.82	17.07	11.17	6.1
**7**	0.8	1.44	1.87	6.29	8.55	17.84	9.12	6.01
**8**	0.35	1.59	1.81	6.44	8.9	17.66	10.06	5.78
**9**	0.63	1.35	2.03	6.16	9.85	17.35	9.46	5.97
**10**	0.79	1.22	2.42	6.08	10.98	16.53	8.8	5.34
**15**	0.34	1.96	2.37	7.6	10.81	16.27	7.56	4.27
**20**	0	2.08	2.89	8.08	11.61	15.81	7	4.55
**25**	0.35	1.69	3.75	7.99	12.15	16.11	6.86	4.34
**30**	0.04	2.4	2.8	10.39	10.84	16.62	7.14	4.14
**35**	0.25	2.19	3.92	8.93	12.82	15.32	7.22	4.41
**40**	1.09	1.58	4.33	9.83	12.81	15.02	7.22	4.53
**45**	0.32	2.39	4.16	10.35	12.79	14.97	7.67	4.32
**50**	0.77	1.85	4.92	9.69	13.58	14.7	7.69	4.72
**55**	1.64	1.39	4.81	10.16	13.09	16.11	6.45	5.38
**60**	1.32	1.84	4.51	10.85	12.15	17.3	5.92	5.62
**70**	1.67	2.02	4.9	10.92	12.45	16.59	5.69	5.48
**263**	7.6	6.14	10.57	9.63	7.77	5.55	0	5.75
**443**	11.67	7.72	13	6.41	8.49	2.31	0	6.13
**624**	13.56	10.1	12.7	3.35	7.76	0	0	6.64
**804**	15.35	11.76	10.86	4.23	4.8	0	0	6.66
**1367**	13.73	13.73	5.38	3.41	2.44	0	0	7.2
**2400**	16.66	9.59	7.95	1.28	2.62	0	0	6.8

Table S5. Second mass spectrometry measurements carried out at 10 ˚C. First part: evolution of the DNA$_2$-[^{107}Ag$_{16-x}$^{109}Ag$_x$Cl$_2$]$^{8+}$ ($0 \leq x \leq 8$) isotopologues over time. Second part: evolution of the DNA$_2$-[^{107}Ag$_{16-x}$^{109}Ag$_x$Cl$_2$]$^{8+}$ ($9 \leq x \leq 16$) isotopologues over time.

**10 °C**		**25 °C**		**40 °C**	
**Time (min)**	**Weighted average**	**Time (min)**	**Weighted average**	**Time (min)**	**Weighted average**
**0**	15.70	**0**	15.70	**0**	15.70
**1**	14.39	**1**	14.00	**1**	14.13

**2**	14.18
**3**	14.04
**4**	13.94
**5**	13.89
**6**	13.81
**7**	13.80
**8**	13.70
**9**	13.71
**10**	13.67
**11**	13.62
**31**	13.07
**51**	12.56
**71**	12.19
**91**	11.96
**111**	11.72
**231**	10.55
**352**	10.11
**472**	9.87
**592**	9.61
**713**	9.61
**893**	9.46
**1074**	9.44
**1254**	9.45
**1435**	9.42
**1615**	9.45
**1976**	9.34
**2336**	9.35

**2**	13.71
**3**	13.40
**4**	13.21
**5**	13.04
**6**	12.85
**7**	12.64
**8**	12.54
**9**	12.45
**10**	12.27
**15**	11.59
**20**	11.03
**25**	10.81
**30**	10.49
**35**	10.25
**40**	10.12
**45**	10.02
**50**	9.93
**55**	9.86
**60**	9.86
**70**	9.76
**80**	9.68
**965**	9.36

**2**	13.60
**3**	12.98
**4**	12.41
**5**	11.88
**6**	11.36
**7**	10.82
**8**	10.48
**9**	10.29
**10**	10.00
**11**	9.87
**13**	9.55
**15**	9.53
**17**	9.40
**19**	9.36
**25**	9.38

Table S6. Time evolution of weighted average $^{107}\text{Ag}_{<16\text{-}8>}$ at 10, 25 and 40 ˚C. The values calculated at 10 ˚ C are from the first mass spectrometry measurements. These values are used in Figure 4 in the manuscript.

Temperature (˚C)	A_1	t_1 (min)	A_2	t_2 (min)	y_0
10	1.73	0.91	4.49	158.48	9.46
25	1.57	0.37	4.48	17.89	9.66
40	6.47	5.41	-	-	9.12

Table S7. Fit results of the curves shown in Figure 4 in the manuscript, where the weighted average $^{107}\text{Ag}_{<16\text{-}8>}$ values are plotted as a function of time. The curves obtained for 10 and 25 ˚C are best fitted with biexponential decay functions, whereas the curve for 40 ˚C is best fitted with a monoexponential decay model.

6. HPLC Chromatograms

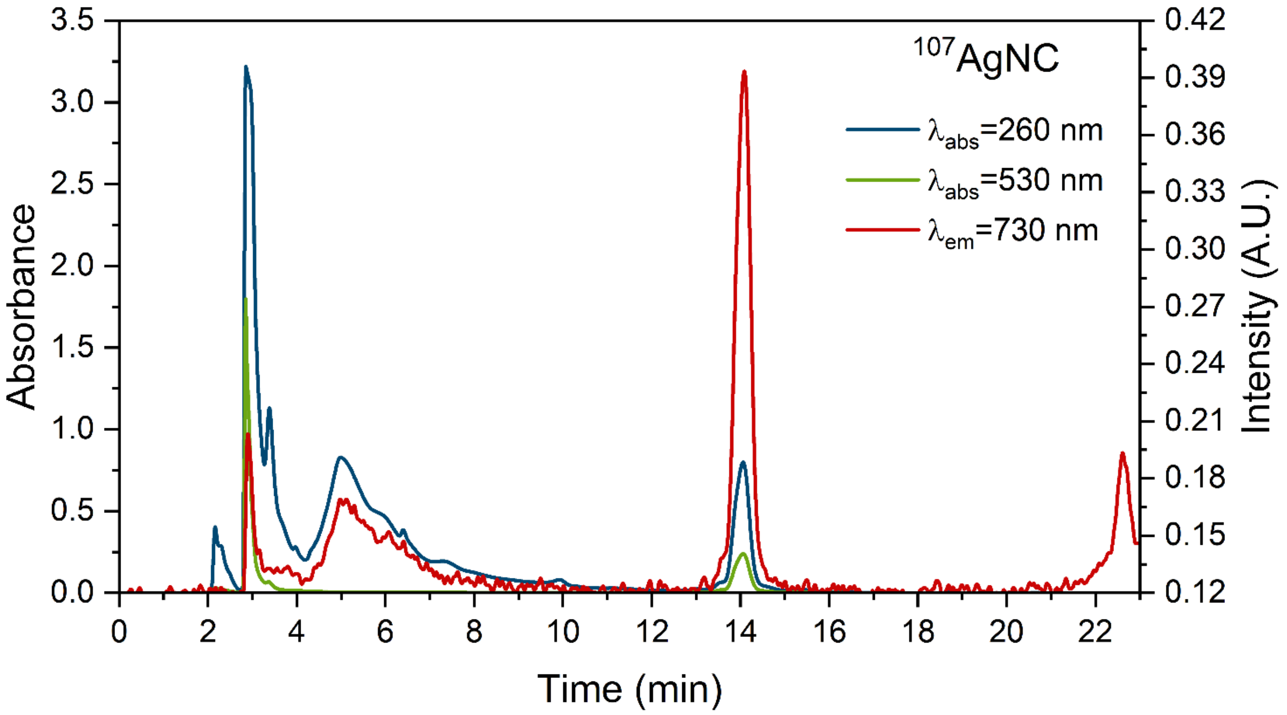

Figure S3. HPLC chromatograms of DNA2-[$^{107}Ag_{16}Cl_2$]$^{8+}$ monitoring the main AgNC absorption peak at 530 nm, monitoring the DNA absorption at 260 nm, and monitoring the emission of the DNA-AgNCs at 730 nm (λ_{exc}= 530 nm). The fraction was collected between 13.5 and 14.5 min.

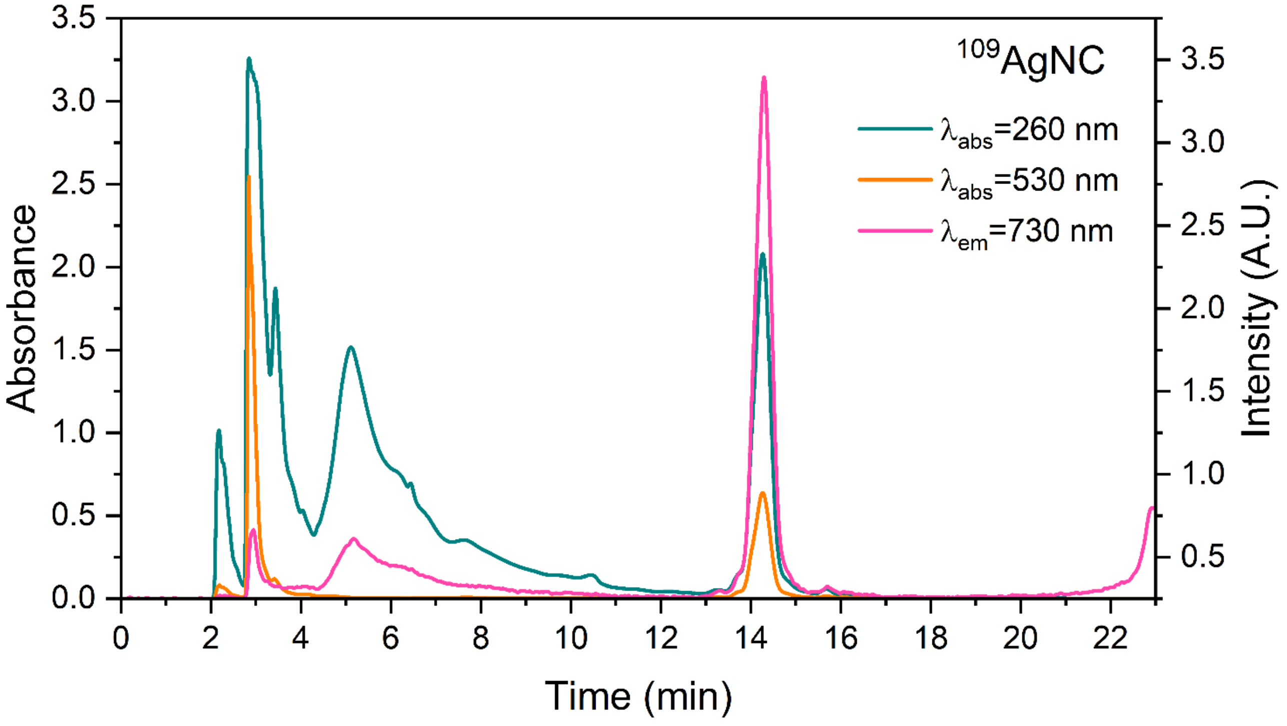

Figure S4. HPLC chromatograms of DNA2-[$^{109}Ag_{16}Cl_2$]$^{8+}$ monitoring the main AgNC absorption peak at 530 nm, monitoring the DNA absorption at 260 nm, and monitoring the emission of the DNA-AgNCs at 730 nm (λ_{exc}= 530 nm). The fraction was collected between 13.5 and 14.5 min.

7. Spectroscopic data

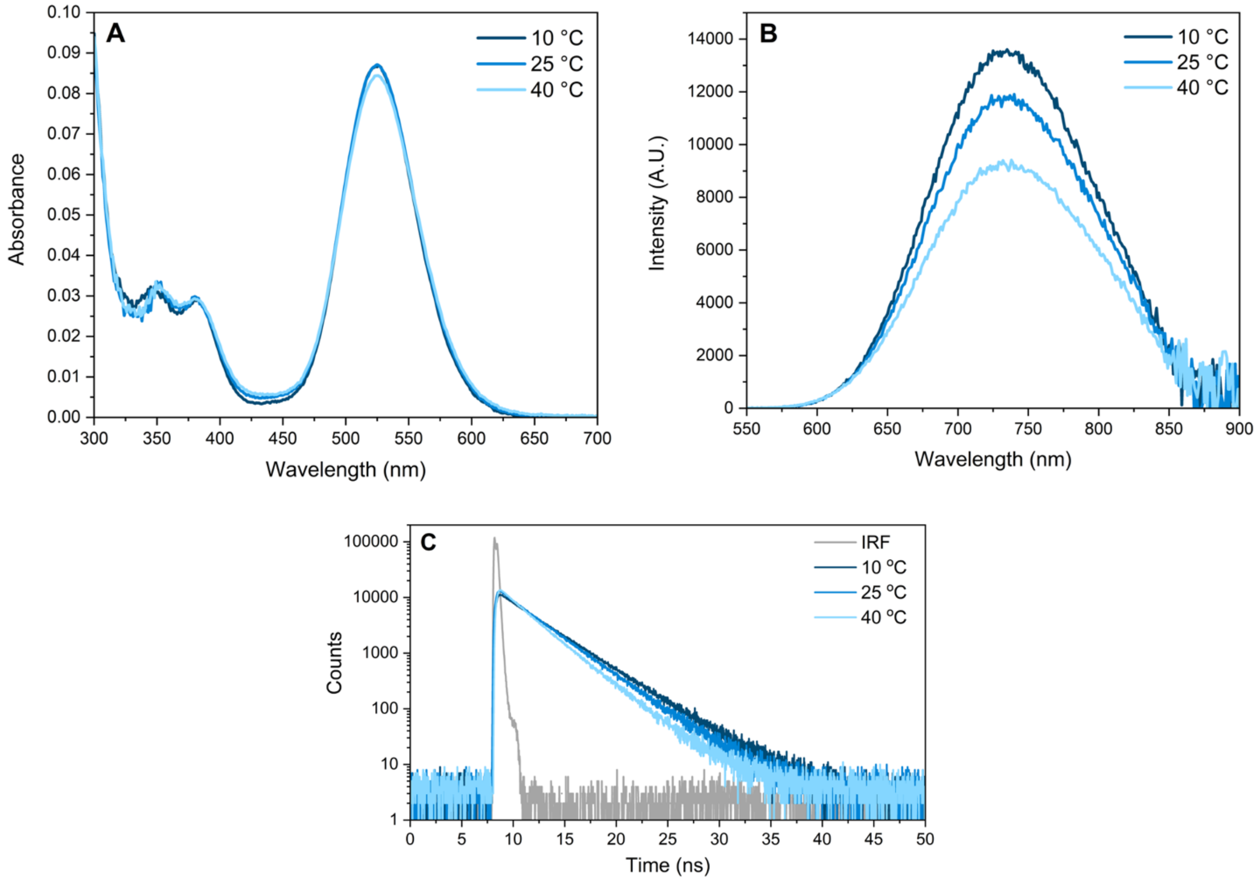

Figure S5. A) Absorption and **B)** emission spectra of natural DNA$_2$-[Ag$_{16}$Cl$_2$]$^{8+}$ in 10 mM ammonium acetate at 10, 25 and 40 °C. **C)** Fluorescence decay curves (λ_{em}=730 nm) of natural DNA$_2$-[Ag$_{16}$Cl$_2$]$^{8+}$ in 10 mM ammonium acetate at 10, 25 and 40 °C. The emission spectra and decays were measured exciting at 531 nm.

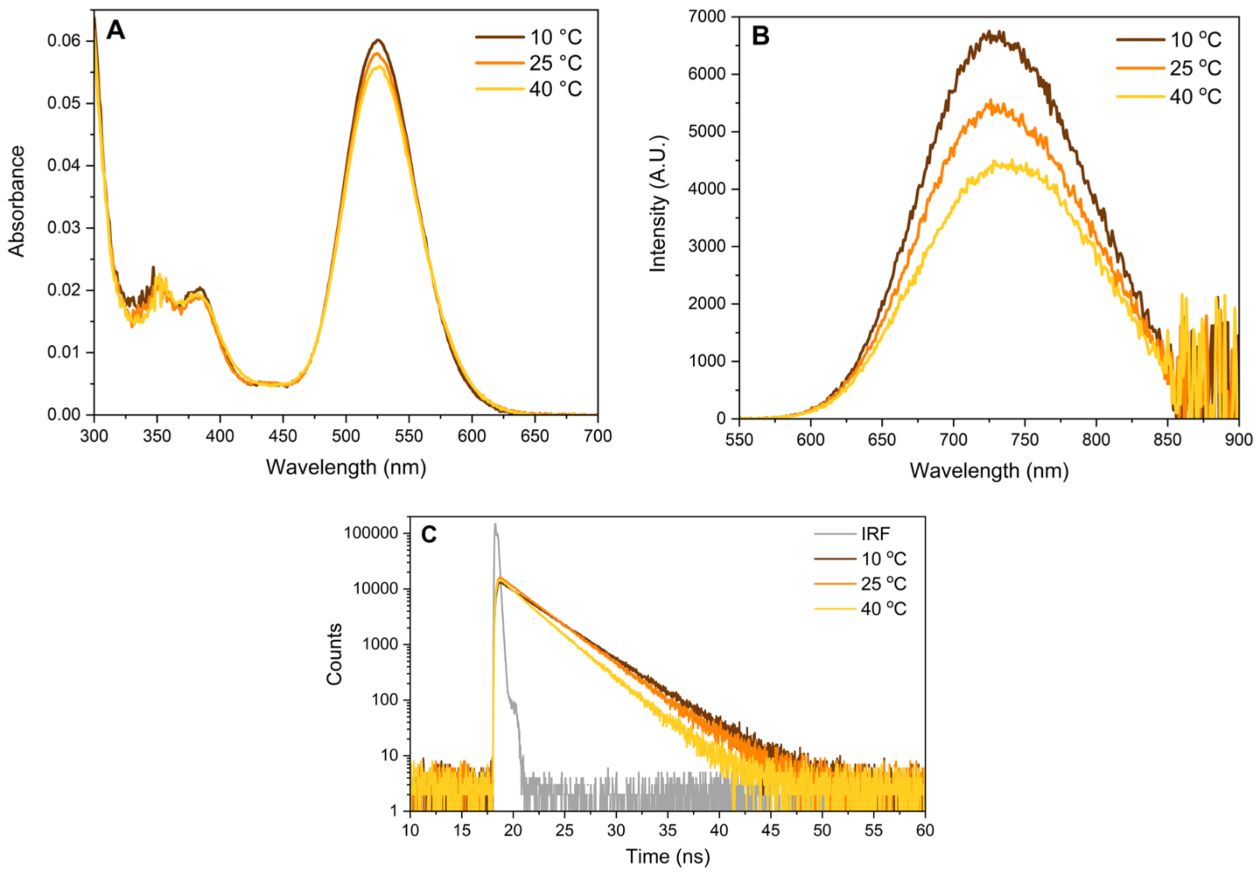

Figure S6. A) Absorption and **B)** emission spectra of DNA$_2$-[$^{107}Ag_{16}Cl_2$]$^{8+}$ in 10 mM ammonium acetate at 10, 25 and 40 ˚C. **C)** Fluorescence decay curves (λ_{em}=730 nm) of DNA$_2$-[$^{107}Ag_{16}Cl_2$]$^{8+}$ in 10 mM ammonium acetate at 10, 25 and 40 ˚C. The emission spectra and decays were recorded exciting at 531 nm.

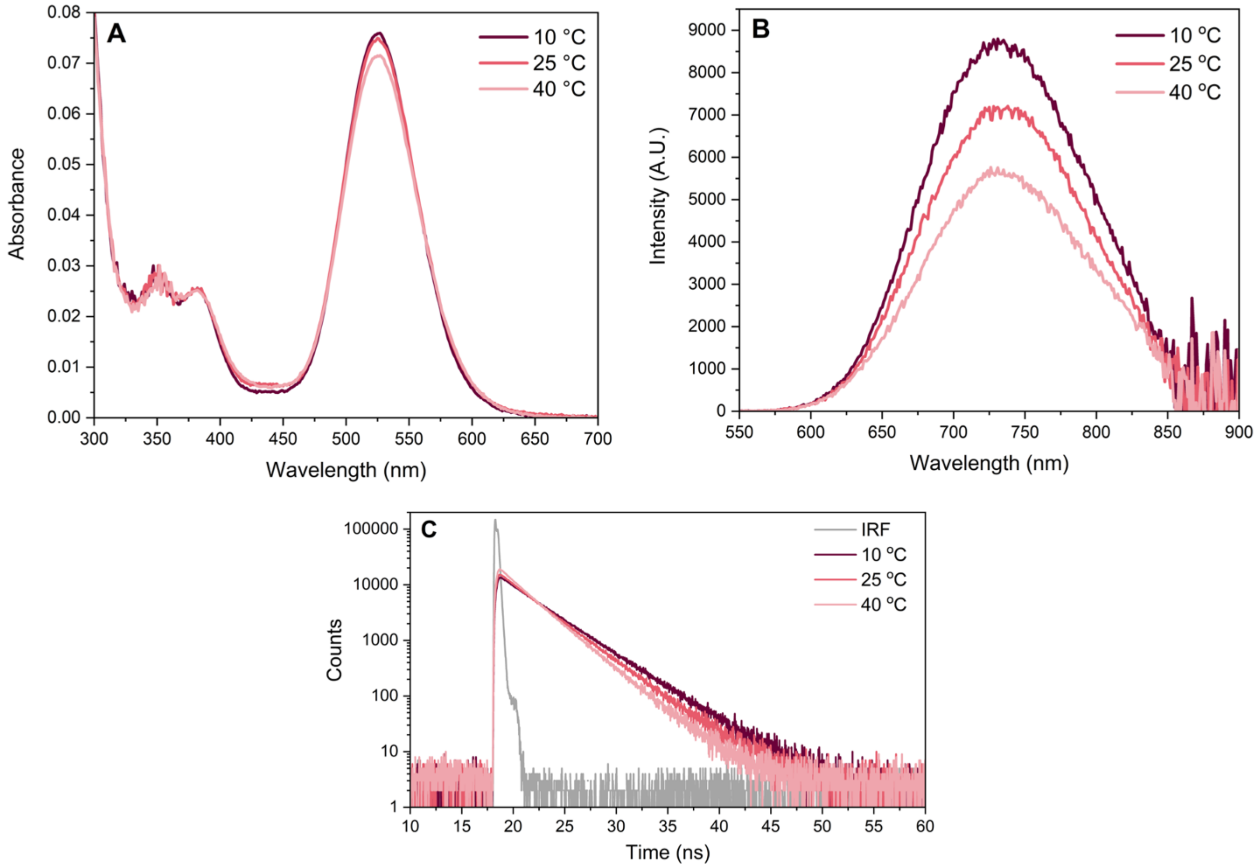

Figure S7. A) Absorption and **B)** emission spectra of DNA_2-$[^{109}Ag_{16}Cl_2]^{8+}$ in 10 mM ammonium acetate at 10, 25 and 40 ˚C. **C)** Fluorescence decay curves (λ_{em}=730 nm) of DNA_2-$[^{109}Ag_{16}Cl_2]^{8+}$ in 10 mM ammonium acetate acquired at 10, 25 and 40 ˚C. The emission spectra and decays were measured exciting at 531 nm.

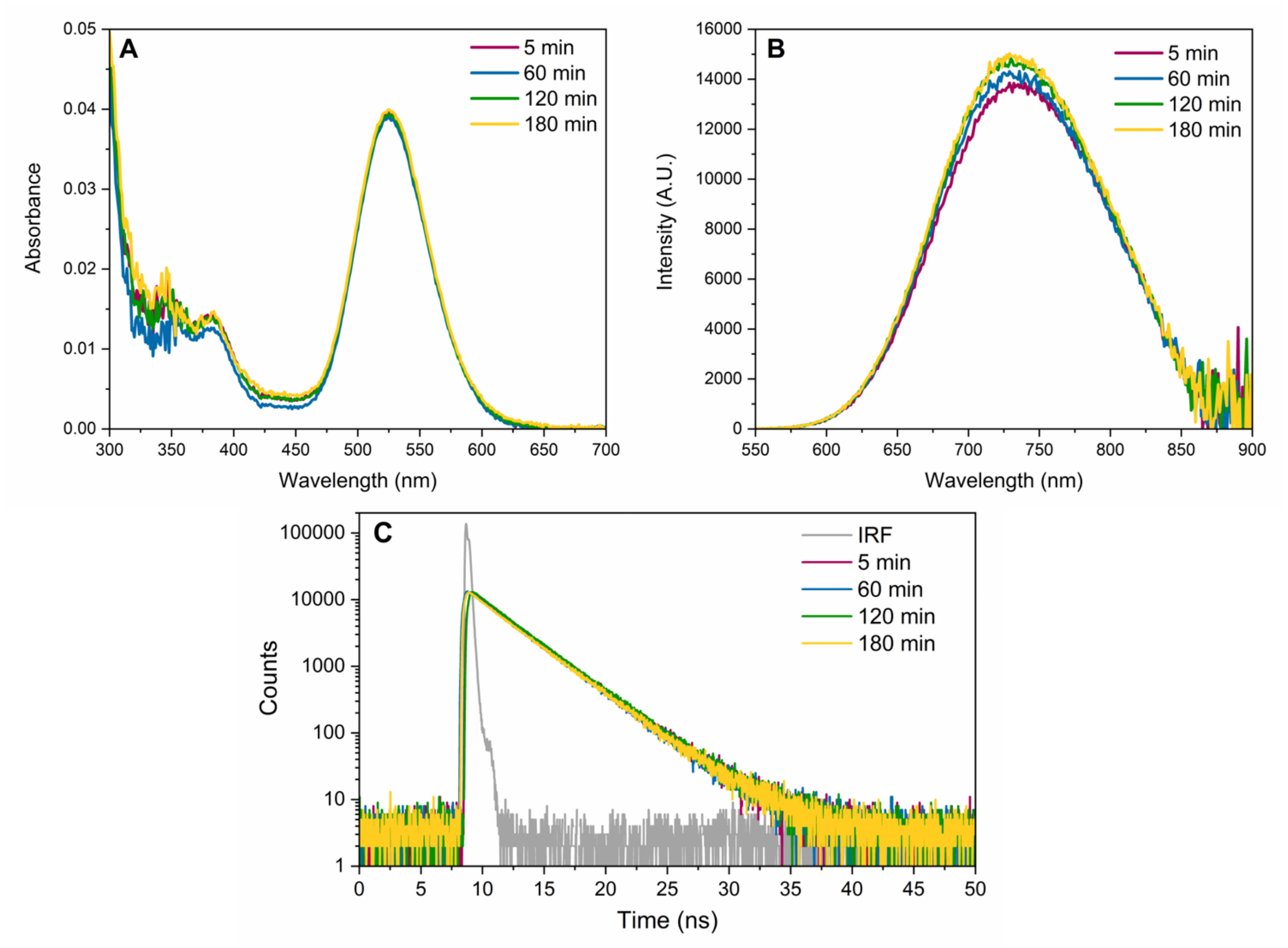

Figure S8. A) Absorption and **B)** emission spectra of a 1:1 mixture of DNA$_2$-[^{107}Ag$_{16}$Cl$_2$]$^{8+}$ and DNA$_2$-[^{109}Ag$_{16}$Cl$_2$]$^{8+}$ in 10 mM ammonium acetate at 10, 25 and 40 ˚C. **C)** Fluorescence decay curves (λ_{em}=730 nm) of the mixture in 10 mM ammonium acetate recorded at 10, 25 and 40 ˚C. The emission spectra and decays were measured exciting at 531 nm.

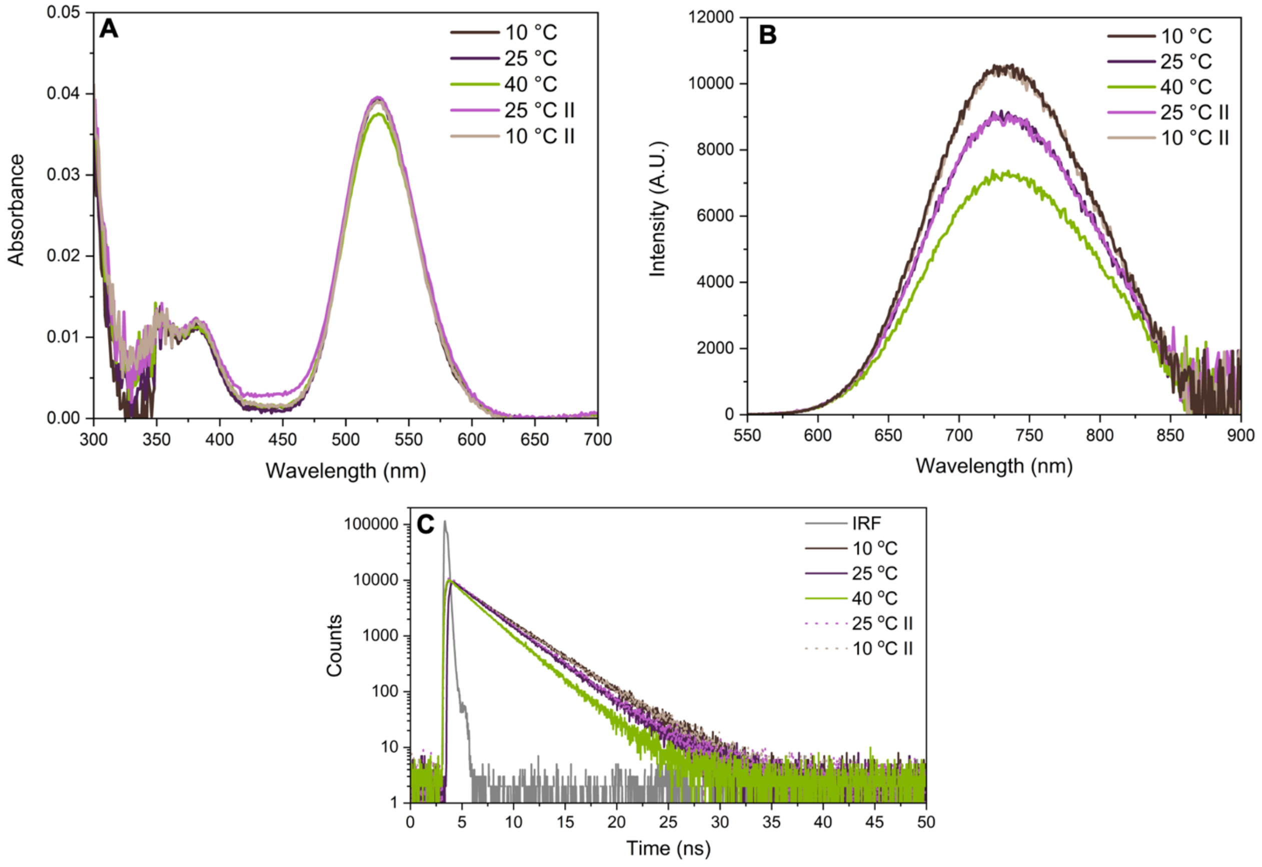

Figure S9. A) Absorption and **B)** emission spectra of a 1:1 mixture of DNA$_2$-[^{107}Ag$_{16}$Cl$_2$]$^{8+}$ and DNA$_2$-[^{109}Ag$_{16}$Cl$_2$]$^{8+}$ in 10 mM ammonium acetate. **C)** Fluorescence decay curves (λ_{em}=730 nm) of the mixture in 10 mM ammonium acetate. The absorption and emission spectra, as well as the decay curves were measured at 10, 25, 40 ˚C and cycled back to 25 and 10 ˚C (indicated with II). The emission spectra and fluorescence decays were measured exciting at 531 nm.

8. Mass spectra

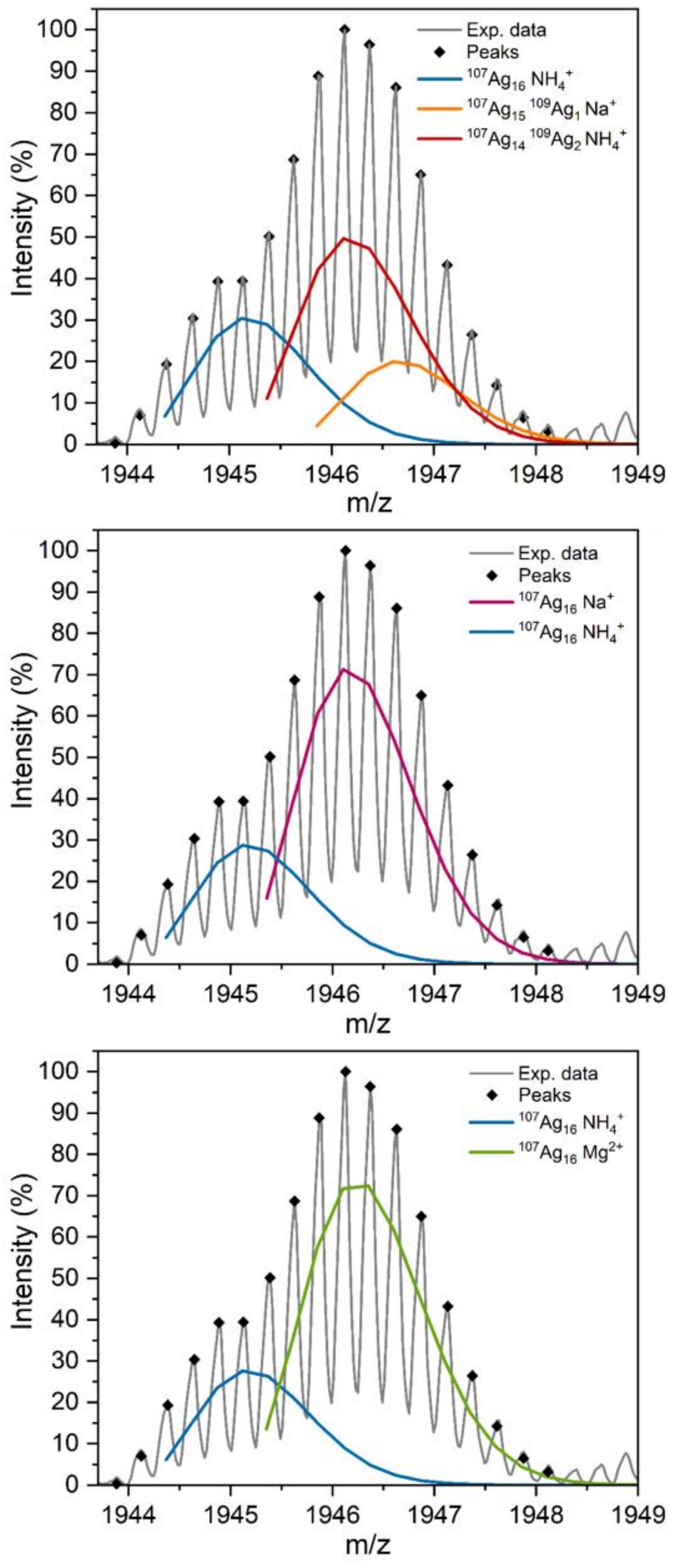

Figure S10. Possible deconvolution analyses for the peak related to DNA-^{107}AgNC adducts (t=0 min).

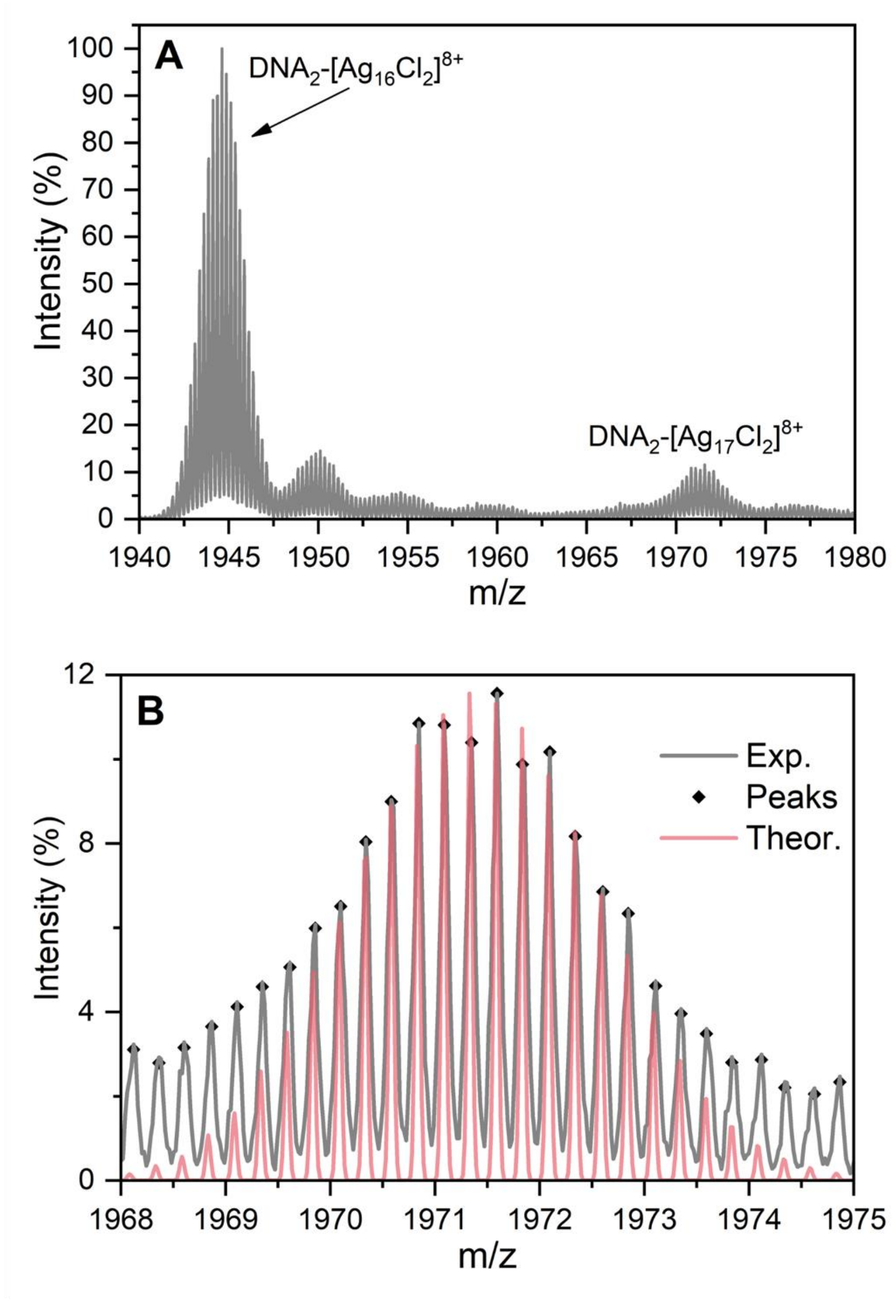

Figure S11. Mass spectrum of DNA_2-$[Ag_{16}Cl_2]^{8+}$ with natural isotopic abundances of silver. **A)** z=4$^-$ peaks and **B)** zoomed-in DNA_2-$[Ag_{17}Cl_2]^{9+}$ peak with the corresponding theoretical isotopic distribution and local maxima.

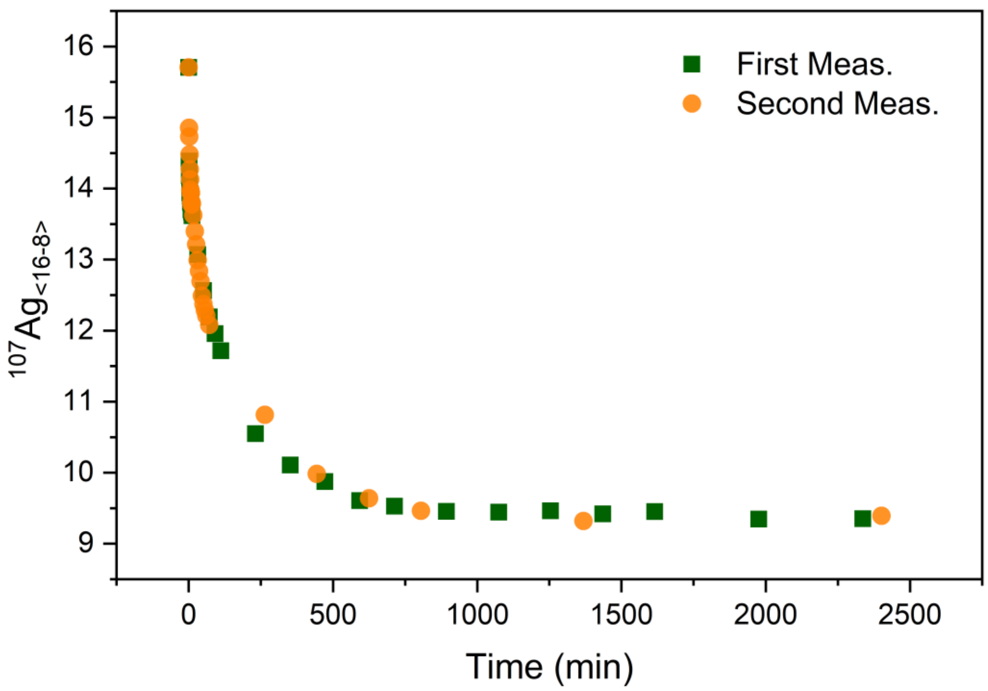

Figure S12. Time evolution of $^{107}Ag_{<16-8>}$ at 10 ˚C; comparison between the first and the second mass spectrometry measurements.

References

1. S. A. Bogh, M. R. Carro-Temboury, C. Cerretani, S. M. Swasey, S. M. Copp, E. G. Gwinn and T. Vosch, *Methods and Applications in Fluorescence*, 2018, **6**, 024004.
